# An Extensive Set of Accurate Fluoride Ion Affinities for *p*‐Block Element Lewis Acids and Basic Design Principles for Strong Fluoride Ion Acceptors

**DOI:** 10.1002/cphc.202000244

**Published:** 2020-04-20

**Authors:** Philipp Erdmann, Jonas Leitner, Julia Schwarz, Lutz Greb

**Affiliations:** ^1^ Anorganisch-Chemisches Institut Ruprecht-Karls-Universität Heidelberg Im Neuenheimer Feld 270 69126 Heidelberg Germany

**Keywords:** benchmark, fluoride ion affinity, Lewis acids, Lewis acidity scale, p-block element compounds

## Abstract

The computed fluoride ion affinity (FIA) is a valuable descriptor to assess the Lewis acidity of a compound. Despite its widespread use, the varying accuracy of applied computational models hampers the broad comparability of literature data. Herein, we evaluate the performance of selected methods (like DLPNO‐CCSD(T)) in FIA computations against CCSD(T)/CBS data and guide for the choice of suitable density functionals that allow the treatment of larger Lewis acids. Based on the benchmarked methods, we computed an extensive set of gas‐phase and solvation corrected FIA, that is covering group 13–16 elements featuring moderate to strong electron‐withdrawing substituents (190 entries). It permits an unbiased comparison of FIA over a significant fraction of the periodic table, serves as a source of reference for future synthetic or theoretical studies, and allows to derive some simple design principles for strong fluoride ion acceptors. Finally, the manuscript includes a tutorial section for the computation of FIA with and without the consideration of solvation.

## Graphical abstract

**A benchmark** for theoretical fluoride ion affinities (FIA) is presented, as well as the best‐performing methods used for the FIA‐computation of a self‐consistent set for 190 p‐block Lewis acids. Trend analysis of the data allowed to derive basic design principles for the construction of strong fluoride ion acceptors.

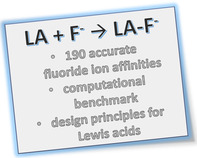

## 1 Introduction

**Figure 1:**
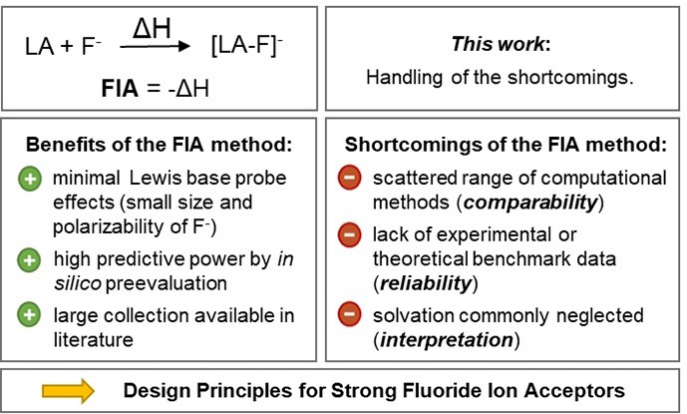
Benefits of the fluoride ion affinity (FIA) as a measure of hard Lewis acidity and its shortcomings as a motivation for the present work (LA=Lewis acid).

Lewis acids play a vital role in all branches of chemistry, today more than ever.^1^ Lewis acidity, the thermodynamic acceptor strength of a Lewis acid, strongly determines its efficiency in bond activation or catalysis. Lewis acidity can be gauged by a variety of experimental or theoretical methods.^2^ Most of those metrics rely on the energetic or spectroscopic output caused by the binding of a probe Lewis base to the Lewis acid of interest. The predominantly applied scale is the fluoride ion affinity (FIA) – the negative enthalpy of the gas phase reaction between a fluoride ion and a Lewis acid (Figure 1).^3^ The small size and polarizability of the fluoride anion minimizes steric repulsion and keeps second‐order effects such as charge transfer, *π*‐back‐bonding, or dispersion as little as possible. Considering the fluoride anion as a *hard* Lewis base, the FIA provides a number that majorly reflects *hard* Lewis acidity.^4^ Since the experimental determination of FIA is nontrivial and requires specialized equipment, it is usually obtained by quantum theoretical computation.2a Hundreds of values have been computed and used meanwhile, e. g., to evaluate the stability of weakly coordinating anions.2a, 5 If conducted correctly, the computational FIA‐method is endowed with a distinct predictive power that allows for the *in silico* preselection of high‐potential candidates. However, the FIA‐method also has severe deficiencies (Figure 1). First, all previous computations have been performed on a wide range of theoretical sophistication, spanning from semi‐empirical to highly correlated *ab initio* models, with or without isodesmic anchoring. As errors exceeding 100 kJ mol^−1^ are possible, this prevents unequivocal comparability. Second, the lack of experimental data impedes a benchmark of the computed FIA and derogates the reliability of the theoretical results. Benchmark studies on the accuracy of FIA computations have been presented only marginally.^6^ A third flaw of the FIA transpires when it comes to the interpretation of solution‐phase experimental data. Since the fluoride ion binding reaction necessarily involves charged species, it is strongly affected by solvation energies – both for cationic and neutral Lewis acids.2a, 5, 7 However, FIA have been computed commonly in vacuum only. In the present work, we attempt to handle the mentioned shortcomings.^8^ In the first part, the DLPNO‐CCSD(T) method and selected density functionals are benchmark for FIA computations against canonical CCSD(T)/CBS reference data. The second part provides an extensive set of 190 computed FIA for literature‐known and hypothetical *p*‐block element‐based Lewis acids, both in the gas‐phase and under consideration of solvation. The presented data is restricted to the common Lewis acidic *p*‐block elements: group 13/14/15 in their highest oxidation states as well as group 15, oxidation state +III, and group 16, oxidation state +IV. Potential aggregation phenomena (e. g., Al_2_F_6_→2 AlF_3_) are not taken into account.^5^ At first, this collection intends to serve as source of reference for correlations of FIA with other theoretical/experimental properties or as inspiration for future synthetic efforts. However, some general trends and maxima allow to extract empirical design principles for the construction of potent fluoride ion acceptors, which are attractive for numerous applications. Finally, a tutorial description for the computation of FIA is given.

## 2 Results and Discussion

The geometries for all involved compounds were optimized with the threefold‐corrected PBEh‐3c/def2‐mSVP composite method, which reaches the structural accuracy of triple‐ζ basis set second‐order perturbation theory (MP2/TZ) across the periodic table.^9^ Different VSEPR and non‐VSEPR structures were considered as starting points, compared in energy, and the lowest energy conformers selected for the final single‐point computations. For ligands with the ability of polydentate binding (e. g., OTf), several coordination isomers were prescreened, and the lowest energy isomers considered further. All optimized structures were verified as local minima by frequency calculations. ZPEs and thermal corrections at 298.15 K were obtained from the PBEh‐3c computations, as implemented in ORCA 4.1.2 or 4.2.^10^ For the subsequent single point energy computation, a benchmark was conducted for a representative set of smaller Lewis acids. As reference data, non‐isodesmic FIA were computed by coupled‐cluster theory with single and double excitations, including perturbative triples correction, CCSD(T). The basis set incompleteness error was treated by a two‐point extrapolation scheme to the complete basis set (CBS) with aug‐cc‐pVnZ (n=3,4) (Table 1, first column).^11^ The obtained values are in agreement with the few known CCSD(T) derived FIA, and reproduce the experimental values of COF_2_ (208.8 kJ mol^−1^) and AlF_3_ (481.2 kJ mol^−1^) with better than chemical accuracy (<4 kJ mol^−1^).6b, 12 To validate the CCSD(T)/CBS method further, the challenging bond dissociation energy of F_2_, as well as the ionization potential and electron affinity of the F‐atom was computed, and compared against experimental data (“F‐test”, Table 2).^13^ These values are reproduced in excellent accuracy, providing additional confidence for the applicability of the CCSD(T)/CBS data as reference and anchor in all following comparisons.

**Table 1:** Benchmark of methods for FIA computation. All values (except column CCSD(T)/CBS and line COF_2_) anchored against TMS‐ref. system, in kJ  mol^−1^. ^a^Non‐isodesmic reference data; ^b^n=normal, t=tight, ^c^T=def2‐TZVPP, Q=def2‐QZVPP; ^d^non‐isodesmic calculation, experimental value: 208.8 kJ mol^−1^, COF_2_ results not considered in MAD/RMSD.

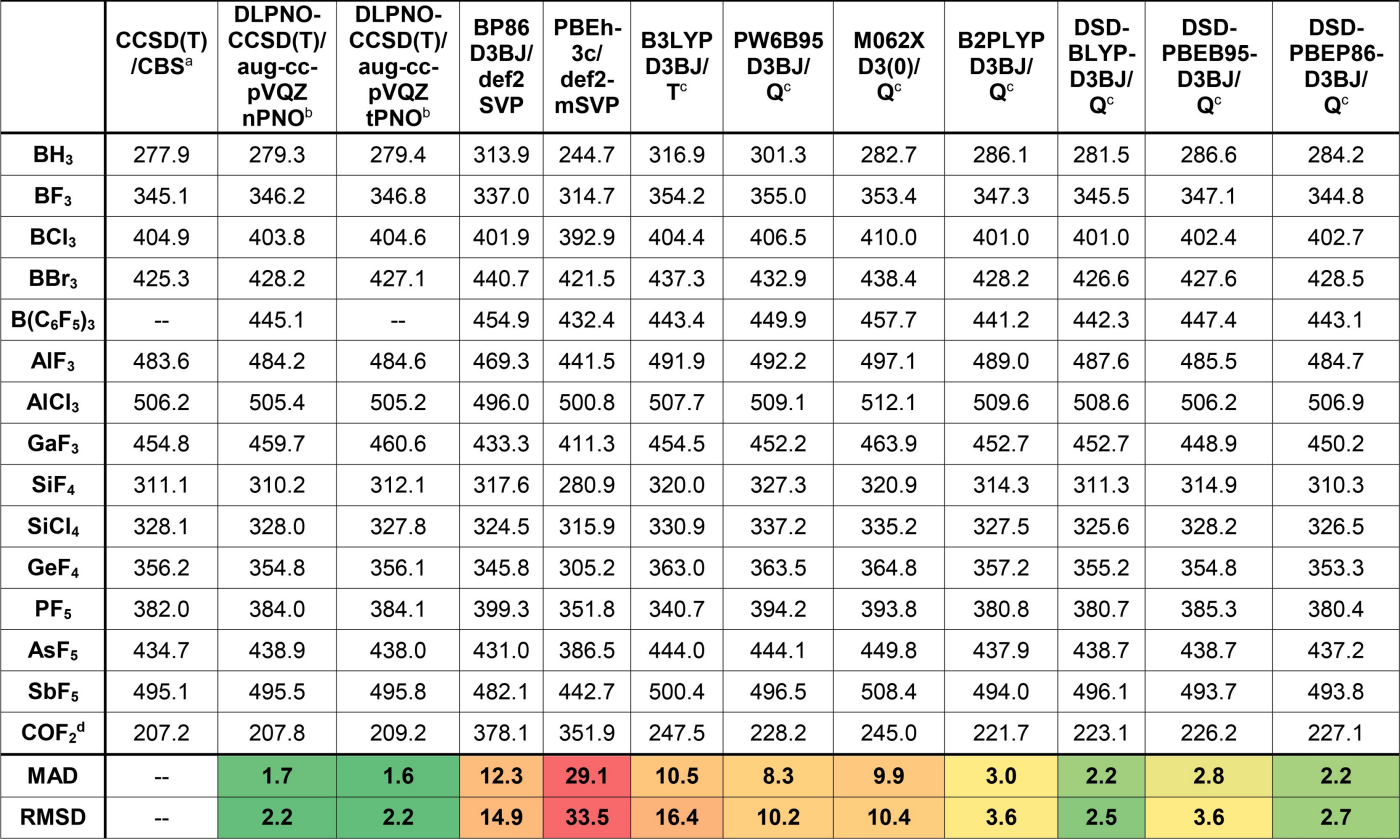

**Table 2:** Computed “*F‐test*”: F_2_‐dissociation enthalpy, electron affinity (EA) and ionization potential (IP) of F‐atom and experimental data. All values in kJ mol^−1^. ^a^T=def2‐TZVPP, Q=def2‐QZVPP.

	CCSD(T)/ CBS	DLPNO‐ CCSD(T)/ aug‐cc‐pVQZ nPNO	DLPNO‐CCSD(T)/ aug‐cc‐pVQZ tPNO	BP86 D3BJ/ def2 SVP	PBEh‐3c/ def2‐mSVP	B3LYP D3BJ/ T^a^	PW6B95 D3BJ/ Q^a^	M062X D3(0)/ Q^a^	B2PLYP D3BJ/ Q^a^	DSD‐ BLYP‐ D3BJ/ Q^a^	DSD‐PBEB95‐D3BJ/ Q^a^	DSD‐PBEP86‐D3BJ/ Q^a^	exp.^13^
BDE F_2_	157.7	150.7	152.2	220.5	106.9	151.9	149.2	138.4	148.4	142.8	146.3	144.3	158.7
EA	−331.2	−327.2	−327.3	−133.5	−123.1	−269.1	−301.2	−296.4	−306.8	−304.6	−291.8	−299.2	−328.2
IP	1680.2	1674.1	1673.7	1692.4	1673.7	1697.4	1696.5	1689.3	1685.8	1681.2	1669.7	1670.5	1681.0

**Figure 2:**
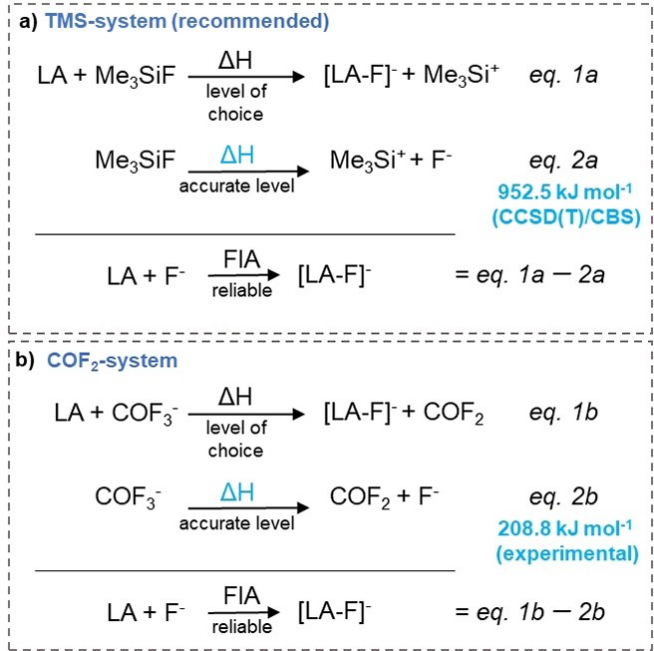
Isodesmic reactions for FIA computation and two established anchors, together with the revised anchor for eq. 2a.

Generally, for all the lower‐level methods, FIA computations via (pseudo‐)isodesmic reactions are mandatory (Figure 2, eqs. 1a/b).3a, 6b As anchor points, the TMS‐system, initially proposed by Krossing et al. (Figure 2a) and the COF_2_‐system (Figure 2b) are established.6b First, the enthalpy of eqs. 1a or 1b is computed at a “level of choice”. Subtraction of the enthalpies of eqs. 2a/b from eqs. 1a/b, respectively, provides the final, absolute FIA. The enthalpies for eqs. 2a/b have to be very exact and are either obtained from top edge level computation or experiment. In that way, the problematic treatment of the “naked” fluoride ion at the (usually low) “level of choice” (applied in eqs. 1) is avoided. At first, the selection of one over the other reference systems (COF_2_ or Me_3_Si^+^) seems now arbitrary. However, the final FIA will depend on how well the “level of choice” in eqs. 1 treats the anchor compounds (COF_2_/COF_3_^−^ or Me_3_Si^+^/Me_3_SiF). Compact anions, such as COF_3_^−^, are more difficult to compute than neutral or cationic species, given that a higher charge density requires a more robust consideration of dynamic electron correlation. By consequence, the computation of eq. 1b is more prone to errors as eq. 1a. This is indeed verified by the better performance of the TMS‐anchored DFT‐data in comparison to the COF_2_‐anchored DFT‐data (see table S3). The TMS‐system anchor energy (eq. 2a) has initially been computed with the Gaussian G3^14^ composite method.6b However, neither G3 (214 kJ mol^−1^) nor G4^15^ (203 kJ mol^−1^) reproduced the non‐isodesmic FIA of COF_2_ as accurate as the above described CCSD(T)/CBS protocol (see table S2). Hence, we revised the TMS‐system anchor energy (eq. 2a) by CCSD(T)/CBS. A slightly lower value of 952.5 kJ mol^−1^ in comparison to the previous G3‐derived value6b (958.4 kJ mol^−1^) was obtained and used for the present study.^16^

Having set up the reference data and specified the isodesmic anchoring, a benchmark of quantum‐theoretical methods applicable to larger Lewis acids was conducted. The local approximation method DLPNO‐CCSD(T), in combination with the aug‐cc‐pVQZ basis and matching auxiliary basis, anchored to the TMS‐system, was benchmarked first (Table 1, column 2).^17^ To our delight, this method reproduced the canonical CCSD(T) data with excellent accuracy (MAD=1.7 kJ mol^−1^). Indeed, with this method, even the challenging F‐test set was treated with good outcome (Table 2). Although the use of tightPNO settings increased the accuracy slightly (Table 1, column 3), we decided to use the substantially faster normalPNO settings as sufficiently accurate for our purpose.^18^ Moreover, neither the use of basis set extrapolation,^19^ decontracted basis sets, the recently introduced iterative computation of triples,^20^ nor the explicitly correlated F12‐basis sets^21^ did show significant improvement that would legitimate the increased computational costs or limitations connected with those modifications. Thus, the DLPNO‐CCSD(T)/aug‐cc‐pVQZ level of theory was identified as an ideal compromise between accuracy and computational cost and chosen as standard model *L1* for small‐to‐medium‐sized Lewis acids in the following collection.

Although the DLPNO‐CCSD(T) method is considered as linear scaling with the size of the system, memory requirements rise quickly and make the computation of huge, polyhalogenated groups, pervasive in modern Lewis acid chemistry, unsuitable. Thus, DLPNO‐CCSD(T) with the non‐augmented cc‐pVQZ basis set was considered for the next size‐scale of molecules. It was assumed, that the neglect of diffuse functions is acceptable for larger anions due to the more efficient spread of the negative charge across the extended system. Indeed, DLPNO‐CCSD(T) FIA computations for a model Lewis acid B(C_6_F_5_)_3_ with aug‐cc‐pVQZ (445 kJ mol^−1^) or the cc‐pVQZ (448 kJ mol^−1^) basis sets revealed an acceptable difference. Thus for the medium‐to‐large‐sized Lewis acids, this model *L2* (DLPNO‐CCSD(T)/cc‐pVQZ) was used in the following collection.

To treat even the largest Lewis acids at a reasonable time and resource costs, selected density functional/basis set combinations were evaluated (Table 1, columns 4 to 12). The benchmark set was extended by the DLPNO‐CCSD(T)/aug‐cc‐pVQZ derived FIA of B(C_6_F_5_)_3_. Again, absolute values were obtained by isodesmic anchoring against the TMS‐system (Figure 2a). Remarkably, although performing overall not best in the series, the low‐cost BP86(D3BJ)/def2‐SVP rendered sufficient for preliminary screening, in line with previous studies.6b Nevertheless, FIA with significantly lower MAD against the CCSD(T)/CBS reference set were obtained with double‐hybrid functionals in combination with the large basis set def2‐QZVPP, in particular with DSD‐BLYP(D3BJ).^22^ The excellent results with double‐hybrid functionals are in agreement with more general benchmarks,^23^ and underline their supremacy also for the particular case of FIA computations. Thus, the DSD‐BLYP(D3BJ)/def2‐QZVPP level of theory granted access to accurate FIA with less computational resources as for the DLPNO‐CCSD(T) method and was used as “low‐cost” model *L3* for the largest Lewis acids in the following collection.

Noteworthily, the M06‐2X(D3Zero)^24^ functional performed moderate with the TMS‐reference system, but revealed a proper matching with the COF_2_‐system (see table S3). Indeed, good results of the M06‐2X functional were also reported in a recent computational study on the energetics of classical Lewis donor‐acceptor interactions.^25^ Importantly, although the best DFT methods are not dramatically inferior compared to DLPNO‐CCSD(T) for the *isodesmic* FIA computation, they absolutely fail in the *non‐isodesmic* FIA computations (line COF_2_ in Table 1, and table S4). Errors up to 170 kJ mol^−1^ (>80 % of total enthalpy!) are possible if FIA are calculated non‐isodesmically. The difficulties of the DFT methods to treat F‐species correctly was further illustrated by the *F‐test* (Table 2). To conclude the benchmark, the ideal overlap of the three identified levels of theory (*L1*‐*L3*) permit for the first time a self‐consistent comparison of multiple FIA spanning several orders of molecular size‐scales. Moreover, this section emphasizes the absolute requirement for isodesmic reactions in case of “low‐cost” computations.

Having identified the most suitable models, a comprehensive and systematic screening of FIA for literature known and hypothetical Lewis acids was performed.^26^ In Table 3, a collection of FIA values can be found, ordered by central elements (group 13: B, Al, Ga; group 14: Si, Ge, Sn; group 15: P, As, Sb). Table 4 contains group 15 and group 16 element compounds in medium oxidation states as well as a selection of special Lewis acids that have been described in the literature. Depending on the molecular size, the values are obtained on the level of theory *L1*‐*L3* and anchored to the TMS‐reference system. Besides the gas‐phase FIA, also the solvation corrected FIA_solv_ values can be found in tables 3/4. For FIA_solv_, the solvation energies of the Lewis acids, the fluoride adducts, and the fluoride anion were obtained with COSMO‐RS^27^ in CH_2_Cl_2_ as implemented in the ADF program package.^28^ The final solvation corrected FIA_solv_ is obtained by eq. (3):

(3) $$\mathrm{FIA}{}_{\mathrm{solv}}=\mathrm{FIA}-[\Delta E{}_{\mathrm{solv}}\left(\mathrm{LA}\text{-}F{}^{-}\right)-\Delta E{}_{\mathrm{solv}}\left(\mathrm{LA}\right)-\Delta E{}_{\mathrm{solv}}\left(F{}^{-}\right)]$$

**Table 3:** Collection of computed FIA for group 13–15 element compounds in highest oxidation states, according models *L1‐L3*, anchored by the TMS‐system, ordered by acceptor element. Color coded acceptor strength (green: highest/red: lowest).

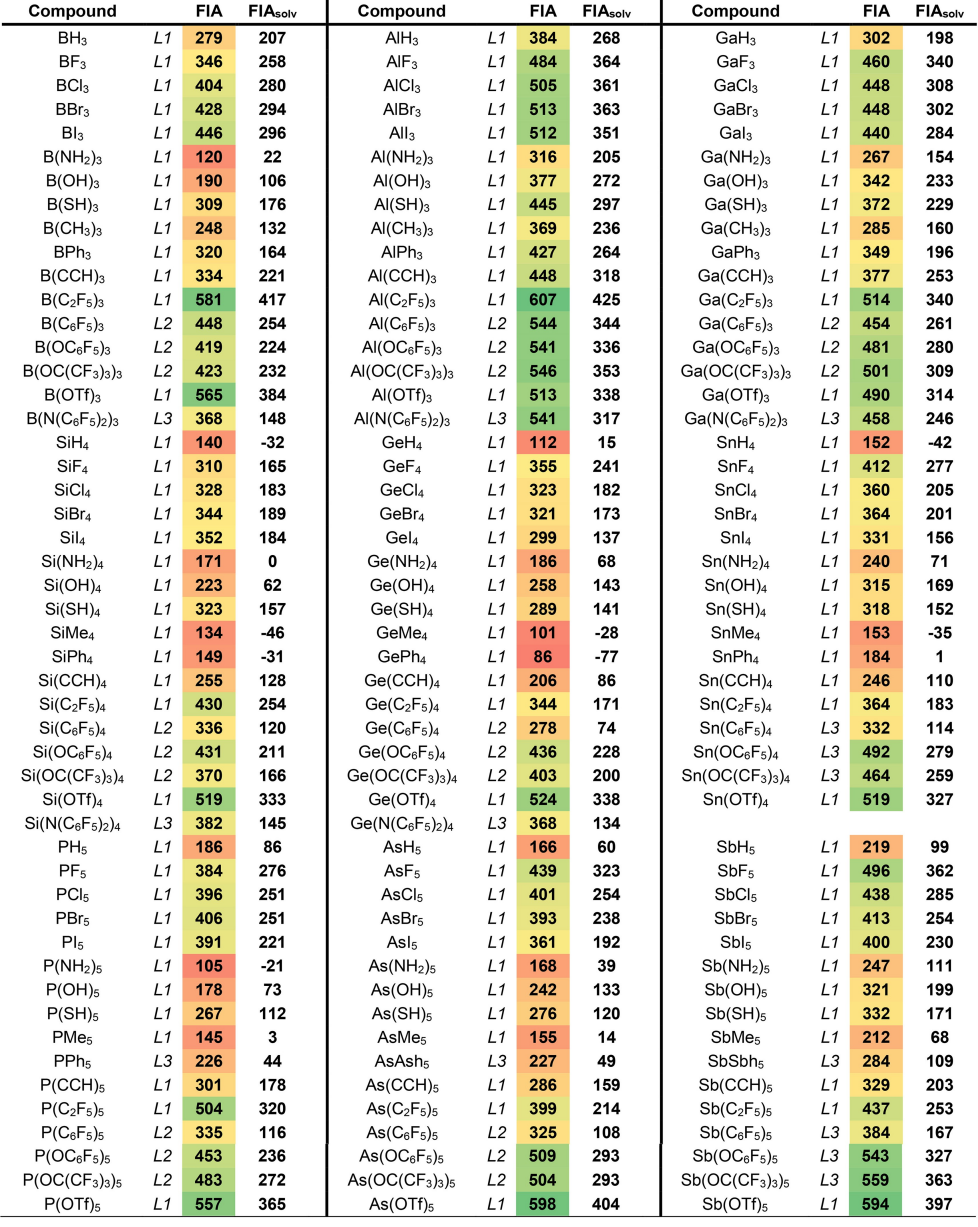

**Table 4:** Collection of computed FIA for group 15–16 element compounds in medium oxidation states, and some selected special Lewis acids, according models *L1‐L3*, anchored by the TMS‐system.

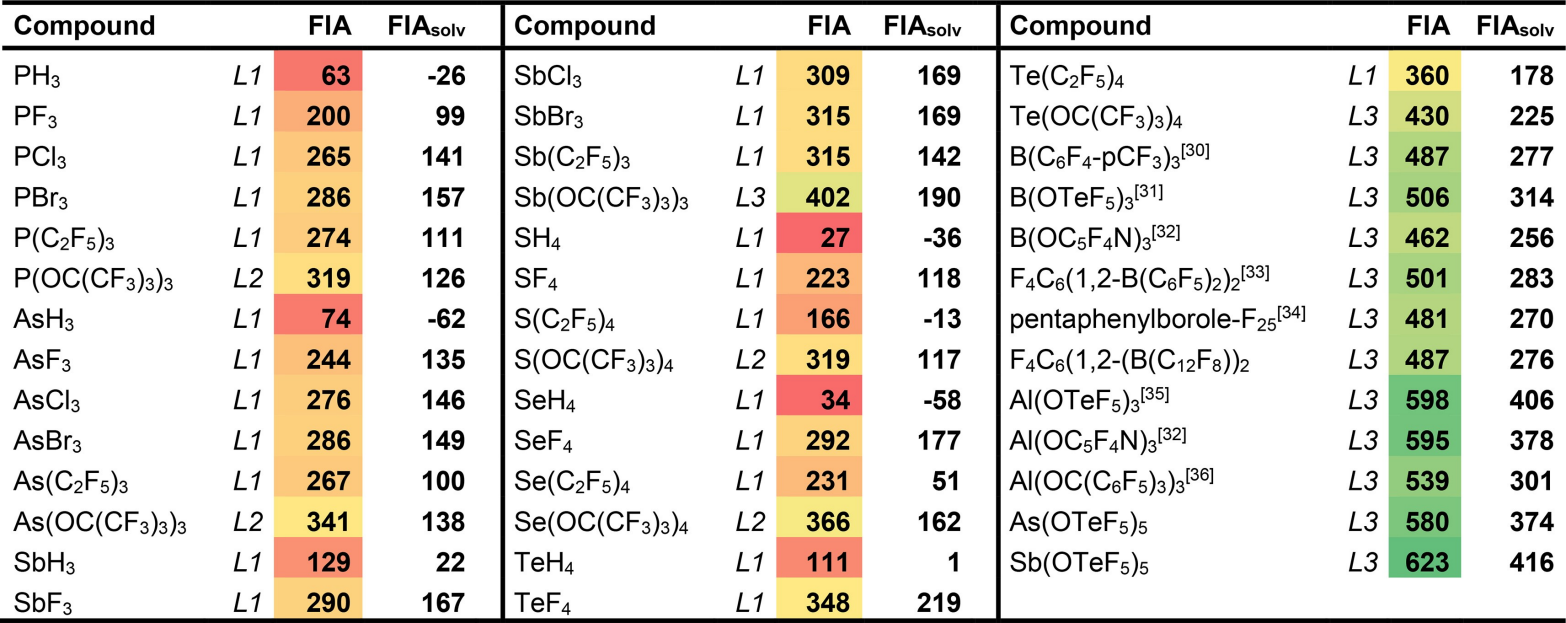

## 3 Discussion

We abstain from an exhaustive discussion of the computed values, as it would branch into a delicate consideration of electrostatic, orbital, steric, and dispersive contribution for every specific Lewis acid. However, by solely focusing on some general trends, the collection allows deriving some basic design principles for the construction of strong fluoride ion acceptors.

**Figure 3:**
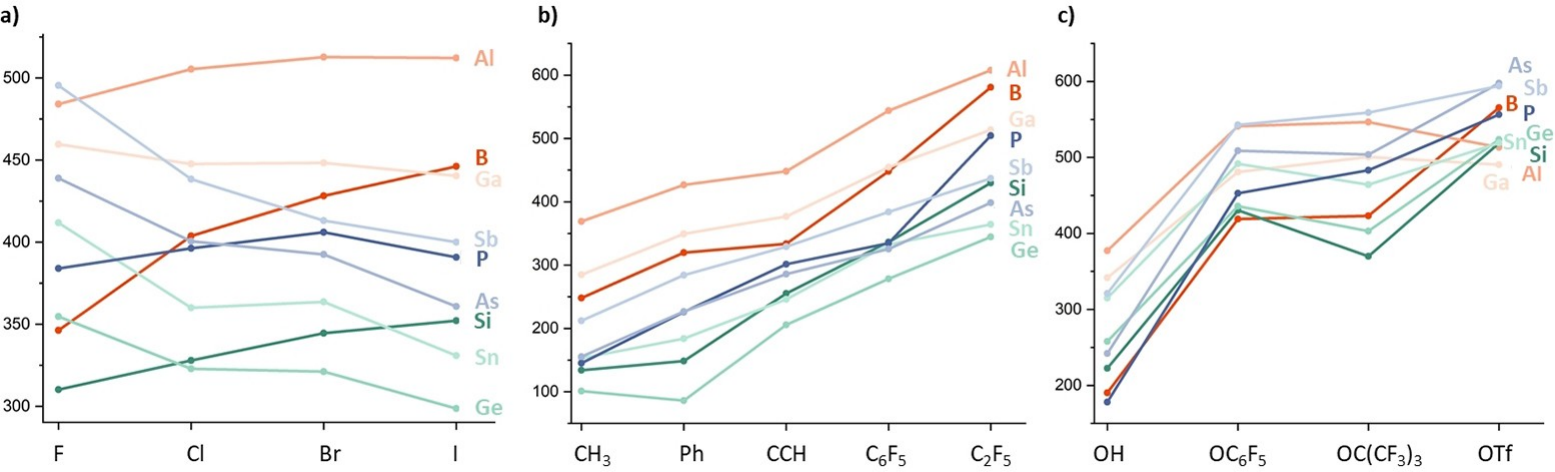
Comparisons of computed FIA for the different ligand classes a) halides b) C‐substituents and c) O‐substituents.

Figure 3a represents the comparison of the FIA for group 13–15 element halides in their highest oxidation states. For the lighter elements (B, Al, Si, and P), the FIA increases in direction F<Cl<Br(< I), whereas for the heavier elements (Ga, Ge, Sn, As, Sb), the opposite trend F>Cl≈Br>I can be found. Interestingly, those trends differ for the lower oxidation state +III in group 15, which generally possess the order F<Cl<Br (see Table 4 and Figure S1). Essentially the same trends remain in solution (FIA_solv_), although dampened by roughly 150 kJ mol^−1^. The findings of this correlation allow to formulate: *In their highest oxidation states, maximum FIA are obtained for light element acceptors with heavy element substituents (e. g., BI_3_) or heavy element acceptors with light element substituents (e. g., SbF_5_)*.

Figure 3b depicts the comparison for the carbon‐based ligands at group 13–15 elements. In contrast to the halide substituents, the trends are less divergent. For the non‐fluorinated C‐ligands, an increase in FIA for sp_3_<sp_2_<sp hybridized carbon ligands is found, in line with their increasing group electronegativity. The prominent C_6_F_5_ group is effective for FIA boosting, but with the C_2_F_5_ substituent the strongest fluoride ion acceptors are obtained. Interestingly, the effect of C_2_F_5_ is most pronounced for the most electronegative elements B and P in this series (*vide infra* for discussion).

Figure 3c illustrates the trends observed with groups connected via oxygen at group 13–15 elements. With the hydroxyl‐substituent, the FIA ordering within each group is similar to the fluoride ligand, although overall diminished. Upon endowing the oxygen atom with a fluorinated aryl or alkyl group, the FIA increases considerably. Although the OC_6_F_5_ group and the OC(CF_3_)_3_ group are roughly in the same “league” of efficiency, a notable difference occurs. The central element determines the change in FIA upon going from OC_6_F_5_ to OC(CF_3_)_3_. Group 13 and group 15 elements experience an increase in FIA (OC(CF_3_)_3_ > OC_6_F_5_), whereas the group 14 elements show a decrease in FIA upon changing OC_6_F_5_ to OC(CF_3_)_3_. With the triflate ligand, B, Si, and Ge outperform Al and Ga for the first time, as for those two latter elements, the free Lewis acids experience a stabilization by bidentate binding of the triflate substituent. The same effect causes the relatively low FIA of Sb(OTf)_5_.

By consideration of the last two comparisons (carbon and oxy substituents), some interesting statements can be made. For the carbon‐based ligands, the σ‐electron‐withdrawing effect is most decisive, since carbon itself is not very electronegative, but has no free electron pairs available to delocalize to the central element. Thus, the C_2_F_5_ group is most effective, especially for the less electropositive elements. For oxy‐ligands, free electron pairs are available, which may delocalize to the central atom and diminish the FIA. The sensitivity of these effects depends on the group and the electronegativity of the central element. The OC_6_F_5_ ligand offers a low‐lying *π*‐system for the free electron pair to delocalize also in the opposite direction. For group 14 species, it is the *π*‐electron density that matters more, in line with previous findings for bis(catecholato)silanes.^29^ For group 13 and 15 elements, especially for the most electronegative P and B, the σ‐electron‐withdrawing effect gains more weight, and the OC(CF_3_)_3_ becomes more effective. As a rule of thumb, one may formulate: *Group 14 acceptors with oxy‐ligands profit from negative mesomeric effects (π‐electron acceptors at oxygen, OC_6_F_6_, perhalogenated catecholates). Group 13 and group 15 acceptors, the more electronegative B and P in particular, profit from negative inductive effects (substituents with electron‐poor* σ‐*framework connected, OC(CF_3_)_3_). For the less electronegative carbon substituents, inductive electron‐withdrawal is most effective in all cases (e. g., C_2_F_5_)*.

A graphical comparison of the computed FIA with the global electrophilicity indices^37^ (based on the PBEh‐3c FMO energies of the free Lewis acids) has been performed, but no correlation was observable (see Figure S2). Last, we would like to comment on the effect of solvation. The COSMO‐RS corrected values substantiate the trend that has been indicated in a previous contribution.2a Overall, the FIA_solv_ is decreased by roughly 100–200 kJ mol^−1^ in comparison to the gas‐phase value. It originates from the larger ΔE_solv_(F^−^) relative to the ΔE_solv_(LA) and ΔE_solv_(LA‐F^−^). The size of the Lewis acid determines the absolute magnitude of this FIA‐damping. Smaller Lewis acids (e. g., BF_3_) lead to compact anions (e. g., BF_4_^−^) with a significant ΔE_solv_(LA‐F^−^) due to high charge density. For larger Lewis acids (e. g., Al(OC(C_6_F_5_)_3_)_3_), the charge density in the fluoride adducts and hence of ΔE_solv_(LA‐F^−^) is much smaller. Thus, for the larger Lewis acids, the FIA‐damping happens to be more pronounced. Tentative comparisons of the FIA‐damping with simply the number of atoms indicated no meaningful correlation. Parameters like the molecular Van der Waals volume^38^ might be better suited, but are not readily available throughout most parts of the periodic table.

### 3.1 Tutorial Section

Finally, a more detailed description of the computation of FIA will be given. It might appear trivial for the experienced scientists in the field, but merits presentation, given some found misuses of the method.

*First Step*: Optimize the geometry of the Lewis acid and of its fluoride ion adduct by a method that describes the structural characteristics of this class of compounds well enough. The use of PBEh‐3c can be recommended, although other functionals with dispersion correction and sufficient basis sets, like B3LYP‐D3(BJ) or TPSS‐D3(BJ), should perform equally well. For all compounds, perform frequency analysis, check for the absence of imaginary frequencies, keep the geometries and the ZPE/thermal corrections for the next step.

*Second Step*: Compute the single point‐energy for both species with either a well‐benchmarked DF (like PW6B95‐(D3BJ)/def2‐QZVPP and the TMS‐reference system or the M06‐2X(D3zero)/def2‐QZVPP and the COF_2_ reference system) or ideally at a higher level of theory (like DSD‐BLYP(D3BJ)/def2‐QZVPP or DLPNO‐CCSD(T)/(aug)‐cc‐pVQZ). Combine the electronic energies obtained from step 2 with the thermal correction of step 1, to obtain the total enthalpies of the Lewis acids and their fluoride adducts. Isodesmic calculations are mandatory if lower levels of theory are applied and generally recommended (see third step).

*Third Step* (must be performed only once): To obtain the absolute FIA values, optimize the two species of the selected reference system (i. e., COF_2_/COF_3_^−^ or Me_3_SiF/Me_3_Si^+^) with the same method as in step 1 and compute the single point enthalpies with the same method as in step 2. Now, calculate the reaction enthalpy of eqs. 1a/b, Figure 2. Next, subtract the experimental value of the FIA of COF_2_ (208.8 kJ mol^−1^) or the high‐level value for the FIA of Me_3_Si^+^ (952.5 kJ mol^−1^). By doing so, a final absolute FIA with an estimated accuracy of ±5 kJ mol^−1^ should be obtained.

*Fourth Step* (optional): Consider solvation correction with a meaningful model.^39^ The solvation free energy has to be computed for the Lewis acid, the fluoride adduct of the Lewis acid, and the fluoride ion. The solvation energies can then be combined with the FIA from step 3, according to eq. 3, to provide the FIA_solv_ data.

## 4 Conclusion

With the present contribution, we provide a benchmark, that identified three methods for the computation of fluoride ion affinities (FIA) suitable for a broad range of p‐block elements and molecular size levels with an estimated accuracy of ±5 kJ mol^−1^. Based on these methods, a self‐consistent set of 190 FIA was computed. With this collection, we provide a source of reference that allows connecting the FIA with models of chemical bonding or experimental findings in future works. Moreover, it allowed for the first time a reliable large‐scale comparison that disclosed two design principles for the construction of strong fluoride ion acceptors: I) Light elements with heavy element substituents or heavy elements with light element substituents. II) *π*‐acceptors at oxy‐ligands for group 14 and σ‐acceptors at oxy‐ligands for groups 13 and 15.

## Conflict of interest

The authors declare no conflict of interest.

## Supporting information

As a service to our authors and readers, this journal provides supporting information supplied by the authors. Such materials are peer reviewed and may be re‐organized for online delivery, but are not copy‐edited or typeset. Technical support issues arising from supporting information (other than missing files) should be addressed to the authors.

Supplementary
