# Supplementary material for: An Extensive Set of Accurate Fluoride Ion Affinities for p‐Block Element Lewis Acids and Basic Design Principles for Strong Fluoride Ion Acceptors
Source: Chemphyschem. 2020 Apr 20;21(10):987–94. doi: 10.1002/cphc.202000244 (PMC7317340; doi:10.1002/cphc.202000244)
Supplement: Supplementary file 1 — Supplementary [file CPHC-21-987-s001.pdf]

ChemPhysChem

Supporting Information

An Extensive Set of Accurate Fluoride Ion Affinities for *p*-Block Element Lewis Acids and Basic Design Principles for Strong Fluoride Ion Acceptors

Philipp Erdmann, Jonas Leitner, Julia Schwarz, and Lutz Greb*© 2020 The Authors. Published by Wiley-VCH Verlag GmbH & Co. KGaA.
This is an open access article under the terms of the Creative Commons Attribution License, which permits use, distribution and reproduction in any medium, provided the original work is properly cited.

Supporting Information for

1. General details and geometry optimization

All calculations (except G3/G4) have been performed with ORCA 4.1.2 and ORCA 4.2.[1] Geometry optimizations were performed with PBEh-3c/def2-mSVP as implemented in ORCA, using *grid5* settings.[2] All calculated geometries have been confirmed as energetic minima on the potential energy surface by analytical calculation of harmonic frequencies at the PBEh-3c level. In case of negative frequencies >10 cm^{-1}, the geometries were reoptimized with *grid6*, *TightOPT* and *VeryTightSCF* settings. Unscaled ZPEs and enthalpies at 298 K were obtained from the same level of theory by using the rigid-rotor harmonic oscillator (RRHO) approximation,[3] as implemented in ORCA. As starting geometries, VSEPR structures preoptimized with UFF were used. In the case of OTf substituents, also polydentate binding modes were considered and compared in energy. Partially, the energies were pretty close in energy at PBEh-3c (around 5 kJ mol^{-1}) and might change in order at higher levels. However, only the most stable (PBEh-3c) isomer was carried over to the single point computation step.

2. Single point energies with canonical coupled cluster theory (CCSD(T))

As reference data for the benchmark set of small Lewis acids, FIA were computed by coupled cluster theory with single and double excitations and perturbative triples correction (CCSD(T)).[4] The frozen core approximation was used with the default number of core electrons per element (see table 9.9 in ORCA 4.2 manual). Single-determinant dominated wavefunctions in the CCSD(T) computations were secured by evaluation of the T1 diagnostics (< 0.02).[5]

For atoms up to bromine, the correlation consistent basis sets aug-cc-pVnZ (n = T,Q) were used.[6] For heavier atoms (> bromine), ECP-based versions of correlation consistent basis sets with weighted core functions, aug-cc-pwCVnZ-PP (n = T,Q) were used in combination with the def2-ECP effective core potentials.[7]

The CCSD(T) total energies were extrapolated the complete basis set limit (CBS) by using a two-point extrapolation scheme[8] for the adjacent aug-cc-pVnZ level basis sets n = T,Q:

$$E^{\mathrm{CBS}}(X,Y) = \frac{E_{\mathrm{HF}}(X)e^{-\alpha\sqrt{(X)}} - E_{\mathrm{HF}}(Y)e^{-\alpha\sqrt{(Y)}}}{e^{-\alpha\sqrt{(X)}} - e^{-\alpha\sqrt{(Y)}}} + \frac{X^{\beta}E_{\mathrm{C}}(X) - Y^{\beta}E_{\mathrm{C}}(Y)}{X^{\beta} - Y^{\beta}}$$

$E_{\mathrm{HF}}(X)$ = HF energy of basis set with cardinal number X (X = 3 or 4); $E_{\mathrm{C}}(X)$ = correlation energy of basis set X; α = 5.79 and β = 3.05 as calibrated in Ref[8c] A recent study on the HF-dimerization energies fully supports the excellent accuracy of this level of theory for fluoro-containing species.[9]

	E(HF) X = 3	E(HF) X = 4	E(C) X = 3	E(C) X = 4	E(HF) CBS	E(C) CBS	E(total)CBS	E(total)CBS in kJ mol^{-1}	therm. Corr. PBEh-3c in kJ mol^{-1}	CBS enthalpy in kJ mol^{-1}	FIA CCSD(T)/CBS in kJ mol^{-1}
**F^-**	-99.45080702	-99.45746209	-0.29872959	-0.319610582	-99.45925196	-0.334475541	-99.7937275	-262008.4315	6.19	-262002.2392	
**BH_3**	-26.400299	-26.402125	-0.138777	-0.143343	-26.402616	-0.146593	-26.549209	-69704.9	80.6	-69624.35757	
**BH_3F^-**	-125.950095	-125.958959	-0.445094	-0.470682	-125.961343	-0.488899	-126.450242	-331995.1	90.6	-331904.4869	**-277.9**
**BF_3**	-323.329463	-323.353206	-0.885265	-0.949554	-323.359592	-0.995320	-324.354912	-851593.8	45.2	-851548.5951	
**BF_4^-**	-422.912063	-422.941802	-1.184502	-1.269982	-422.949800	-1.330834	-424.280634	-1113948.8	52.9	-1113895.886	**-345.1**
**BCl_3**	-1403.376091	-1403.388346	-0.748288	-0.805082	-1403.391642	-0.845512	-1404.237154	-3686824.6	34.9	-3686789.703	
**BCl_3F^-**	-1502.984975	-1503.003367	-1.042387	-1.121618	-1503.008314	-1.178022	-1504.186336	-3949241.2	44.3	-3949196.875	**-404.9**
**BBr_3**	-7742.183043	-7742.192675	-0.857257	-1.066685	-7742.195265	-1.215774	-7743.411039	-20330325.7	31.9	-20330293.83	
**BBr_3F^-**	-7841.803740	-7841.820128	-1.148328	-1.379264	-7841.824536	-1.543665	-7843.368201	-20592763.2	41.9	-20592721.33	**-425.3**
**Alf_3**	-540.603012	-540.633721	-0.873272	-0.937230	-540.641980	-0.982761	-541.624741	-1422035.8	35.3	-1422000.486	
**AlF_4^-**	-640.243387	-640.281311	-1.165949	-1.251275	-640.291511	-1.312018	-641.603529	-1684530.1	43.8	-1684486.3	**-483.6**
**$AlCl_3$**	-1620.720611	-1620.734917	-0.713445	-0.772735	-1620.738765	-0.814943	-1621.553707	-4257389.3	29.4	-4257359.845	
**$AlCl_3F^-$**	-1720.369194	-1720.391737	-1.005090	-1.086034	-1720.397800	-1.143657	-1721.541457	-4519907.1	38.8	-4519868.267	**-506.2**
**GaF_3**	-2221.854792	-2221.881981	-0.966443	-1.090562	-2221.889293	-1.178921	-2223.068214	-5836665.6	32.5	-5836633.077	
**GaF_4^-**	-2321.482532	-2321.516774	-1.265552	-1.408315	-2321.525984	-1.509947	-2323.035931	-6099130.8	40.7	-6099090.164	**-454.8**
**SiF_4**	-687.153337	-687.194534	-1.173388	-1.259983	-687.205614	-1.321629	-688.527243	-1807728.3	52.1	-1807676.152	
**SiF_5^-**	-786.721379	-786.766901	-1.474355	-1.582678	-786.779144	-1.659792	-788.438936	-2070046.4	56.9	-2069989.494	**-311.1**
**$SiCl_4$**	-2127.244404	-2127.264470	-0.978680	-1.058537	-2127.269867	-1.115386	-2128.385252	-5588075.5	39.3	-5588036.23	
**$SiCl_4F^-$**	-2226.817086	-2226.843398	-1.280482	-1.382158	-2226.850475	-1.454539	-2228.305014	-5850414.8	48.2	-5850366.577	**-328.1**
**GeF_4**	-2473.491751	-2473.527488	-1.264122	-1.404672	-2473.537099	-1.504728	-2475.041828	-6498222.3	44.0	-6498178.345	
**GeF_5^-**	-2573.076730	-2573.118814	-1.564959	-1.726701	-2573.130133	-1.841842	-2574.971976	-6760588.9	52.1	-6760536.797	**-356.2**
**PF_5**	-838.310527	-838.359352	-1.496957	-1.606817	-838.372484	-1.685025	-840.057509	-2205571.0	61.8	-2205509.167	
**PF_6^-**	-937.907816	-937.961244	-1.797513	-1.928555	-937.975613	-2.021843	-939.997456	-2467963.3	69.9	-2467893.39	**-382.0**
**AsF_5**	-2731.710992	-2731.754247	-1.575141	-1.736652	-2731.765880	-1.851631	-2733.617511	-7177112.8	55.5	-7177057.264	
**AsF_6^-**	-2831.331420	-2831.380670	-1.873067	-2.054514	-2831.393916	-2.183685	-2833.577600	-7439558.0	63.8	-7439494.203	**-434.7**
**SbF_5**	-736.741068	-736.780100	-1.930541	-2.098324	-736.790597	-2.217766	-739.008363	-1940266.5	52.1	-1940214.375	
**SbF_6^-**	-836.386354	-836.432334	-2.223319	-2.412240	-836.444700	-2.546730	-838.991431	-2202772.0	60.3	-2202711.684	**-495.1**
**COF_2**	-311.742038	-311.763986	-0.924283	-0.982835	-311.769889	-1.024518	-312.794407	-821241.7	47.2	-821194.4818	
**COF_3^-**	-411.268500	-411.296489	-1.228419	-1.307179	-411.304016	-1.363247	-412.667263	-1083457.9	54.0	-1083403.893	**-207.2**
**Me_3Si^+**	-407.607901	-407.619393	-0.718502	-0.747266	-407.622484	-0.767742	-408.390226	-1072228.5	317.2	-1071911.319	
**Me_3SiF**	-507.424243	-507.444800	-1.013723	-1.063724	-507.450329	-1.099319	-508.549648	-1335197.1	331.0	-1334866.086	**-952.5**

Table S1: CCSD(T) derived energies [in Hartree, except where given] used for the CBS extrapolation and the herewith calculated FIA data (non-isodesmic).

3. Revised TMS-system anchor point

The TMS-system anchor enthalpy of $Me_3Si^+ + F^- \rightarrow Me_3SiF$ and the experimentally known FIA of COF_2 was (re)computed at the G3, G4 and the above described CCSD(T)/CBS level of theory. For the computations with the G3 and G4 composite method, Gaussian 16, Revision C.01 has been used.[10] For the deviation of the CCSD(T)/CBS enthalpies, see table S1.

	**Enthalpy [Hartree]**	**Enthalpy [kJ mol^{-1}]**	**FIA [kJ mol^{-1}]**
**G3**			
**F^-**	-99.80683	-262042.8	
**COF_2**	-312.87769	-821460.4	**-213.9**
**COF_3^-**	-412.76600	-1083717.1	
**Me_3Si^+**	-408.67945	-1072987.9	**-958.5**
**Me_3SiF**	-508.85133	-1335989.2	
**G4**			
**F^-**	-99.83128	-262107.0	
**COF_2**	-312.93936	-821622.3	**-203.4**
**COF_3^-**	-412.84812	-1083932.7	
**Me_3Si^+**	-408.71695	-1073086.4	**-946.7**
**Me_3SiF**	-508.90881	-1336140.1	
**CCSD(T)/CBS**			
**F^-**		-262002.2	
**COF_2**		-821194.5	**-207.2**
**COF_3^-**		-1083403.9	
**Me_3Si^+**		-1071911.3	**-952.5**
**Me_3SiF**		-1334866.1	

Table S2: Comparison of G3/G4 and CCSD(T)/CBS values for the FIA of the reference systems.

The FIA of Me_3Si^+ for the Gaussian G3 and the G4 method differs by roughly 12 kJ mol^{-1}, a point the might stem from the fact that for the updated G4 strategy, the higher level correction terms (HLC) have been redefined and in contrast to the G3 method differentiate between atomic anions and molecules.[11] Of all three levels of theory, the CCSD(T)/CBS computed FIA of COF_2 agrees best with experiment, and led us to use the respective TMS-system anchor value of 952.5 kJ mol^{-1} for the remainder of this work.

4. Single Point Energies with DLPNO-CCSD(T) (*L1* and *L2*)

The single point electronic energies for the compounds in the FIA(CCSD(T)/CBS) benchmark set were calculated with the linear scaling version of domain-based localized pair natural orbitals based coupled cluster theory (DLPNO-CCSD(T)), as implemented in ORCA 4.1.2 or 4.2.[12] Throughout, NormalPNO and the herewith connected threshold settings were used. As shown in table 1 (main part), tightPNO settings improve the results only marginally. Like with canonical CCSD(T), for atoms up to bromine, the correlation consistent basis sets (aug)-cc-pVnZ (n = T,Q) were used.[6] For heavier atoms (> bromine), ECP-based versions of correlation consistent basis sets with weighted core functions, (aug)-cc-pwCVnZ-PP (n = T,Q) were used, including the def2-ECP effective core potentials.[7] To speed up the HF-part, the resolution of identity and "chain of spheres" RIJCOSX approximation (*gridX6*) was used with the respective auxiliary basis sets.[13] Single-determinant dominated wavefunctions in the DLPNO-CCSD(T) computations were secured by evaluation of the T1 diagnostics (< 0.02).[5]

5. Single Point Energies with Density Functional Theory

For the DFT-based benchmark, the following functional/basis set combinations were used on the PBEh-3c geometries/thermal corrections and anchored to the TMS-system (table 1, main part) or COF_2-system (table S3):

BP86[14] + D3(BJ)[15] with def2-SVP[16]

B3LYP[17] + D3(BJ) with def2-TZVPP[16]

PW6B95[18] + D3(BJ) with def2-QZVPP[19]

M06-2X[20] + D3(zero) with def2-QZVPP

B2PLYP[21] + D3(BJ) with def2-QZVPP

DSD-BLYP[22] + D3(BJ) with def2-QZVPP

DSD-PBEB95[23] + D3(BJ) with def2-QZVPP

DSD-PBEP86[24] + D3(BJ) with def2-QZVPP

In all DFT computations, the resolution-of-identity[25] and "chain of spheres"[13] approximation in the form of RIJCOSX was used in combination with matching auxiliary basis sets (def2/J or AutoAux).[26] For the double-hybrid functionals including MP2 correlation, the def2/C auxiliary basis set was applied.

	BP86 D3BJ/ def2 SVP	PBEh-3c/ def2-mSVP	B3LYP D3BJ/ T^c	PW6B95 D3BJ/ Q^c	M062X D3(0)/ Q^c	B2PLYP D3BJ/ Q^c	DSD-BLYP-D3BJ/ Q^c	DSD-PBEB95-D3BJ/ Q^c	DSD-PBEP86-D3BJ/ Q^c
BH_3	313.0	280.5	301.7	287.8	269.0	277.6	277.0	283.8	279.7
BF_3	338.0	350.6	338.9	341.5	339.7	338.9	341.1	344.3	340.3
BCl_3	402.9	428.7	389.2	393.0	396.3	392.6	396.6	399.6	398.2
BBr_3	441.7	457.4	422.1	419.4	424.7	419.8	422.1	424.9	424.0
$B(C_6F_5)_3$	455.9	468.2	428.2	436.4	444.0	432.8	437.8	434.3	438.6
AlF_3	470.2	477.3	476.7	478.7	483.4	480.6	483.1	482.8	480.2
$AlCl_3$	497.0	536.7	492.5	495.6	498.4	501.2	504.1	503.5	502.4
GaF_3	434.2	447.1	439.3	438.8	450.2	444.3	448.3	446.1	445.6
SiF_4	318.5	316.8	304.8	313.8	307.2	305.8	306.9	312.1	305.8
$SiCl_4$	325.4	351.8	315.7	323.7	321.5	319.1	321.2	325.4	322.0
GeF_4	346.8	341.1	347.7	350.0	351.1	348.7	350.8	352.0	348.8
PF_5	400.2	387.6	325.5	380.7	380.1	372.3	376.3	382.6	375.9
AsF_5	431.9	422.3	428.8	430.6	436.1	429.5	434.2	435.9	432.7
SbF_5	483.1	478.6	485.2	483.1	494.7	485.6	491.6	490.9	489.3
MAD	11.9	15.0	14.4	7.3	4.0	7.2	4.2	3.5	5.0
RMSD	14.6	17.9	19.3	8.4	5.0	8.0	4.9	4.7	5.5

Table S3: DFT-computed FIA of benchmark set, anchored by the COF_2-reference system. In comparison to the TMS-system (main part, table 1), less good MAD/RMSD are obtained.

	BP86 D3BJ/ def2 SVP	PBEh-3c/ def2-mSVP	B3LYP D3BJ/ T^c	PW6B95 D3BJ/ Q^c	M062X D3(0)/ Q^c	B2PLYP D3BJ/ Q^c	DSD-BLYP-D3BJ/ Q^c	DSD-PBEB95-D3BJ/ Q^c	DSD-PBEP86-D3BJ/ Q^c
BH_3	482.2	621.7	340.4	307.2	305.2	290.5	291.3	301.2	298.0
BF_3	506.3	423.6	377.6	361.0	375.9	351.7	355.4	361.7	358.6
BCl_3	571.2	611.3	427.9	412.4	432.5	405.5	410.8	417.0	416.5
BBr_3	610.0	493.7	460.8	438.9	460.9	432.6	436.4	442.3	442.3
$B(C_6F_5)_3$	624.2	565.5	466.9	455.8	480.2	445.6	452.1	462.0	456.9
AlF_3	638.5	679.8	515.4	498.2	519.6	493.4	497.4	500.2	498.5
$AlCl_3$	665.3	600.5	531.2	515.1	534.6	514.1	518.4	520.9	520.7
GaF_3	602.5	459.9	478.0	458.2	486.5	457.1	462.5	463.5	463.9
SiF_4	486.8	494.9	343.5	333.3	343.5	318.7	321.1	329.5	324.1
$SiCl_4$	493.7	530.7	354.4	343.2	357.7	331.9	335.4	342.8	340.3
GeF_4	515.1	477.2	386.4	369.4	387.3	361.6	365.0	369.4	367.1
PF_5	568.5	484.2	364.2	400.2	416.3	385.2	390.6	400.0	394.2
AsF_5	600.2	620.5	467.5	450.1	472.3	442.3	448.5	453.3	451.0
SbF_5	651.4	590.3	523.9	502.5	531.0	498.5	505.9	508.3	507.6
MAD	169.0	143.1	30.2	13.9	32.4	5.6	10.1	15.9	13.5
RMSD	169.6	164.1	31.9	15.3	32.6	6.6	10.4	16.2	13.8

Table S4: Non-isodesmic DFT-computed FIA of benchmark set. In comparison to the isodesmic reactions (main part, table 1 and table S3), much less good MAD/RMSD are obtained.

6. Solvation correction with COSMO-RS

The decrease in the free energy of an isolated molecule or ion by the interaction with the solvent can be obtained by several quantum chemical solvation models.[27] We choose the COSMO-RS solvation model, as implemented in ADF[28], based on BP86-D3/TZ2P[29] single point energy calculations for the electrostatic solute-solvent interaction on the PBEh-3c gas-phase geometries.[30] With the COSMO-RS model, accurate solvation free energies with an error of about 1 kcal mol^{-1} for neutral molecules are usually obtained.[27a, 30d] The dependency of the outcome of COSMO-RS on the DFT method has found to be very weak.[31] The validity of predictions of the solvation free energies for charged species is indicated by good performance in the computation of pk_a values in organic solvents.[32] The obtained value ΔG_{solv} reflects equal concentrations in the gas phase and in solution, e.g. the transfer from a 1M ideal gas (24.79 bar) to a 1M ideal solution.[27a, 33]

It should be noted, that the mixing of a solvation *free* energy (ΔG_{solv}) is obtained by most quantum-theoretical solvation models. The FIA on the other hand, is a reaction *enthalpy* (ΔH). Mixing those two values is not strictly correct. However, in contrast to the herein presented $FIA_{(gas)}$ values, the FIA_{solv} values are not intended to be absolute, and in any case strongly affected by the problematic solvation free energy of an isolated fluoride ion. To avoid confusion, in eq. 4 of the manuscript, ΔE_{solv} was written instead of $\Delta G_{solv.}$

Tables of computed FIA

		DLPNO-CCSD(T)/ aug-cc-pVQZ	DLPNO-CCSD(T)/ cc-pVQZ	DSD-BLYP(D3BJ)/ QZVPP
	TMS_Kation	-1071846.28	-1071850.81	-1072693.35
	TMSF	-1334753.89	-1334745.1	-1335720.81
			TMS-anchor	952.5

Method
aug-cc-pVQZ (L1)
cc-pVQZ (L2)
DSD-BLYP (L3)

	fluoride (COSMO-RS)	deltaG_solv kcal	deltaG_solv kJ
		-75.05202	**-314.017652**

Compound	total thermal correction [kJ mol-1]	electr. energy [Hartree] L1/L2/L3	electr. energy [kJ mol-1] L1/L2/L3	electr. Energy + thermal correction	dH (eq 1)	FIA	COSMO-RS	Diff_solv	FIAsolv
							kcal	kJ	
BH3	80.6	-26.54553	-69695.3	-69614.7			0.3		
BH3_F	90.6	-126.42914	-331939.7	-331849.1	673.2	**279.3**	-57.4	72.5	**206.8**
BF3	45.2	-324.30096	-851452.2	-851406.9			-1.2		
BF3_F	52.9	-424.20918	-1113761.2	-1113708.3	606.3	**346.2**	-55.3	87.8	**258.5**
BCl3	34.9	-1404.19189	-3686705.8	-3686670.9			-3.9		
BCl3_F	44.3	-1504.12268	-3949074.1	-3949029.7	548.7	**403.8**	-49.3	123.9	**279.8**
BBr3	31.9	-7743.24973	-20329902.2	-20329870.3			-5.3		
BBr3_F	41.9	-7843.19006	-20592295.5	-20592253.6	524.3	**428.2**	-48.3	134.0	**294.2**
BI3	30.0	-917.08197	-2407798.7	-2407768.7			-7.7		
BI3_F	40.6	-1017.02937	-2670210.6	-2670170.0	506.3	**446.2**	-46.9	149.9	**296.2**
B(NH2)3	243.2	-192.60105	-505674.1	-505430.9			-4.9		
B(NH2)3_F	250.4	-292.42278	-767756.0	-767505.6	832.9	**119.6**	-56.6	97.8	**21.8**
B(OH)3	146.0	-252.24412	-662266.9	-662121.0			-4.6		
B(OH)3_F	150.6	-352.09171	-924416.8	-924266.2	762.4	**190.1**	-59.6	83.9	**106.2**
B(SH)3	109.3	-1219.96042	-3203006.1	-3202896.8			-5.9		
B(SH)3_F	116.7	-1319.85417	-3465277.1	-3465160.5	644.0	**308.5**	-49.4	132.1	**176.4**
B(CH3)3	325.5	-144.35889	-379014.3	-378688.7			-2.4		
B(CH3)3_F	333.0	-244.22958	-641224.8	-640891.7	704.6	**247.9**	-49.7	116.2	**131.7**
BPh3	791.3	-718.63983	-1886788.9	-1885997.6			-9.8		
BPh3_F	795.5	-818.53664	-2149067.9	-2148272.5	632.7	**319.8**	-47.6	155.9	**163.9**
B(CCH)3	188.4	-254.69153	-668692.6	-668504.3			-6.1		
B(CCH)3_F	195.5	-354.59478	-930988.6	-930793.1	618.8	**333.7**	-54.3	112.5	**221.2**
B(C2F5)3	286.9	-1749.62925	-4593651.6	-4593364.7			-4.7		
B(C2F5)3_F	295.3	-1849.62711	-4856196.0	-4855900.7	371.7	**580.8**	-40.7	163.4	**417.5**
B(C6F5)3	498.9	-2205.97423	-5791785.3	-5791286.4			-10.1		
B(C6F5)3_F	503.4	-2305.91493	-6054179.7	-6053676.3	504.4	**448.1**	-38.8	194.1	**254.0**
B(OC6F5)3	546.1	-2431.51717	-6383948.3	-6383402.3			-12.0		
B(OC6F5)3_F	550.5	-2531.44672	-6646313.4	-6645762.9	533.7	**418.8**	-40.5	195.2	**223.7**
B(OC(CF3))3	581.7306	-3400.72741	-8928609.8	-8928028.1			-7.5		
B(OC(CF3))3_F	583.6534	-3500.65769	-9190976.8	-9190393.1	529.2	**423.3**	-36.8	191.1	**232.2**
B(OTf)3	322.7	-2906.59573	-7631267.1	-7630944.4			-10.3		
B(OTf)3_F	331.2	-3006.58778	-7893796.2	-7893465.0	387.0	**565.5**	-42.1	181.3	**384.2**
B(N(C6F5)2)3	1044.3	-4554.17488	-11956986.1	-11955941.8			-16.4		
B(N(C6F5)2)3_F	1047.8	-4654.13557	-12219432.9	-12218385.1	584.1	**368.4**	-38.9	219.9	**148.5**
AlH3	60.3	-243.77214	-640023.7	-639963.4			-2.1		
AlH3_F	69.2	-343.69515	-902371.6	-902302.4	568.7	**383.8**	-49.5	115.7	**268.2**
AlF3	35.2	-541.56923	-1421890.0	-1421854.8			-5.5		
AlF3_F	43.72	-641.53029	-1684337.8	-1684294.1	468.3	**484.2**	-51.8	120.6	**363.6**
AlCl3	29.4	-1621.50692	-4257266.4	-4257237.0			-6.6		
AlCl3_F	38.8	-1721.47641	-4519736.3	-4519697.5	447.1	**505.4**	-47.2	144.3	**361.1**
AlBr3	27.7	-7960.57087	-20900478.8	-20900451.1			-7.5		
AlBr3_F	37.4	-8060.54330	-21162956.4	-21162919.1	439.7	**512.8**	-46.7	150.1	**362.7**
AlI3	26.8	-1134.41498	-2978406.5	-2978379.7			-9.6		
AlI3_F	36.7	-1234.38726	-3240883.7	-3240847.0	440.3	**512.2**	-46.1	161.4	**350.9**
Al(NH2)3	227.6	-409.81590	-1075971.6	-1075744.0			-5.2		
Al(NH2)3_F	234.1	-509.71224	-1338249.5	-1338015.4	636.3	**316.2**	-53.8	110.8	**205.4**
Al(OH)3	128.9	-469.47616	-1232609.7	-1232480.8			-7.1		
Al(OH)3_F	136.9	-569.39636	-1494950.2	-1494813.2	575.2	**377.3**	-56.8	105.7	**271.6**
Al(SH)3	99.5	-1437.23268	-3773454.4	-3773354.9			-8.2		
Al(SH)3_F	108.5	-1537.17912	-4035863.8	-4035755.3	507.2	**445.3**	-47.8	148.0	**297.3**
AlMe3	311.9	-361.57606	-949317.9	-949006.0			-4.1		
AlMe3_F	318.3	-461.49240	-1211648.3	-1211330.0	583.7	**368.8**	-47.4	133.0	**235.8**
AlPh3	780.3	-935.84255	-2457054.6	-2456274.3			-11.6		
AlPh3_F	785.0	-1035.78023	-2719441.0	-2718656.0	526.0	**426.5**	-47.8	162.4	**264.1**
Al(CCH)3	178.9	-471.93456	-1239064.2	-1238885.3			-8.5		
Al(CCH)3_F	185.7	-571.88120	-1501474.1	-1501288.4	504.5	**448.0**	-52.5	129.8	**318.2**
Al(C2F5)3	276.1	-1966.85888	-5163988.0	-5163711.9			-8.2		
Al(C2F5)3_F	283.7	-2066.86659	-5426558.2	-5426274.5	345.0	**607.5**	-39.7	182.0	**425.5**
Al(C6F5)3	486.6	-2423.20777	-6362132.0	-6361645.41			-11.9		
Al(C6F5)3_F	495.9	-2523.18675	-6624626.8	-6624130.95	408.7	**543.8**	-39.2	199.8	**344.0**
Al(OC6F5)3	530.4	-2648.77998	-6954371.8	-6953841.4			-13.8		
Al(OC6F5)3_F	541.1	-2748.75855	-7216865.6	-7216324.4	411.3	**541.2**	-39.9	205.0	**336.2**
Al(OC(CF3))3	570.8626	-3618.00968	-9499084.4	-9498513.6			-8.6		
Al(OC(CF3))3_F	576.6728	-3717.98837	-9761578.5	-9761001.8	406.1	**546.4**	-37.5	193.3	**353.1**
Al(OTf)3	313.5	-3123.92907	-8201875.8	-8201562.3			-11.0		
Al(OTf)3_F	319.5	-3223.90033	-8464350.3	-8464030.8	439.1	**513.4**	-44.1	175.6	**337.8**
Al(N(C6F5)2)3	1031.8	-4771.62748	-12527908.0	-12526876.1			-17.9		
Al(N(C6F5)2)3_F	1039.1	-4871.65539	-12790531.2	-12789492.1	411.5	**541.0**	-39.5	223.7	**317.3**
GaH3	61.2	-1925.28532	-5054836.6	-5054775.5			-0.4		

GaH3_F	69.0	-2025.17687	-5317101.9	-5317032.9	650.2	**302.3**	-50.5	104.3	**198.0**
GaF3	32.5	-2222.96914	-5836405.5	-5836373.0			-4.4		
GaF3_f	40.7	-2322.92074	-6098828.4	-6098787.7	492.8	**459.7**	-51.0	119.3	**340.4**
GaCl3	27.8	-3302.96518	-8671935.1	-8671907.3			-5.5		
GaCl3_F	36.7	-3402.91251	-8934346.8	-8934310.1	504.8	**447.7**	-47.1	140.1	**307.6**
GaBr3	26.3	-9642.04104	-25315178.8	-25315152.4			-6.7		
GaBr3_F	35.6	-9741.98871	-25577591.4	-25577555.8	504.3	**448.2**	-46.8	146.0	**302.2**
GaI3	25.8	-2825.25361	-7417703.4	-7417677.6			-8.8		
GaI3_F	35.1	-2925.19834	-7680108.2	-7680073.1	512.0	**440.5**	-46.4	156.7	**283.8**
Ga(NH2)3	226.1	-2091.27825	-5490651.0	-5490425.0			-5.0		
Ga(NH2)3_F	233.6	-2191.15621	-5752880.6	-5752647.0	685.6	**266.9**	-53.0	113.0	**153.9**
Ga(OH)3	128.1	-2150.90744	-5647207.5	-5647079.4			-6.4		
Ga(OH)3_F	134.7	-2250.81355	-5909511.0	-5909376.3	610.8	**341.7**	-55.4	109.0	**232.7**
Ga(SH)3	99.2	-3118.71713	-8188191.8	-8188092.6			-7.2		
Ga(SH)3_F	106.9	-3218.63502	-8450526.2	-8450419.3	580.9	**371.6**	-48.1	142.6	**229.0**
GaMe3	310.5	-2043.08208	-5364112.0	-5363801.5			-3.2		
GaMe3_F	318.6	-2142.96699	-5626359.8	-5626041.2	667.9	**284.6**	-48.4	124.8	**159.8**
GaPh3	779.6	-2617.34950	-6871851.1	-6871071.5			-10.9		
GaPh3_F	783.8	-2717.25751	-7134159.6	-7133375.8	603.3	**349.2**	-49.2	153.6	**195.6**
Ga(CCH)3	42.0	-2153.42929	-5653828.6	-5653786.6			-7.3		
Ga(CCH)3_F	43.5	-2253.34682	-5916162.1	-5916118.6	575.7	**376.8**	-52.8	123.7	**253.1**
Ga(C2F5)3	275.7	-3648.37236	-9578801.6	-9578525.9			-6.9		
Ga(C2F5)3_F	282.5	-3748.34396	-9841277.1	-9840994.6	438.9	**513.6**	-40.4	173.8	**339.7**
Ga(C6F5)3	487.9	-4104.70186	-10776894.7	-10776406.8			-11.4		
Ga(C6F5)_F	494.3	-4204.64575	-11039297.4	-11038803.1	498.0	**454.5**	-40.1	193.8	**260.7**
Ga(OC6F5)3	527.9	-4330.19872	-11368936.7	-11368408.9			-13.2		
Ga(OC6F5)3_F	538.4	-4430.15421	-11631369.9	-11630831.5	471.7	**480.8**	-40.4	200.5	**280.3**
Ga(OC(CF3))3	568.3964	-5299.42185	-13913632.1	-13913063.7			-8.4		
Ga(OC(CF3))3_F	575.8368	-5399.38376	-14176082.1	-14175506.2	451.7	**500.8**	-37.7	191.3	**309.4**
Ga(OTf)3	309.9	-4805.36572	-12616487.7	-12616177.8			-9.6		
Ga(OTf)3_F	320.3	-4905.32991	-12878943.7	-12878623.4	462.0	**490.5**	-42.5	176.2	**314.3**
Ga(N(C6F5)2)3	1028.4	-6453.69883	-16944186.3	-16943157.9			-15.3		
Ga(N(C6F5)2)3_F	1037.0	-6553.69544	-17206727.4	-17205690.4	495.0	**457.5**	-39.9	211.1	**246.4**
SiH4	94.4	-291.45168	-765206.4	-765112.0			-1.0		
SiH4_F	99.7	-391.28053	-1027307.0	-1027207.3	812.3	**140.2**	-34.9	172.1	**-31.9**
SiF4	52.1	-688.45223	-1807531.3	-1807479.2			-1.6		
SiF4_F	56.9	-788.34564	-2069801.5	-2069744.6	642.3	**310.2**	-42.0	145.1	**165.1**
SiCl4	39.3	-2128.32116	-5587907.2	-5587868.0			-5.0		
SiCl4_F	48.2	-2228.22292	-5850199.3	-5850151.1	624.5	**328.0**	-45.4	145.1	**182.9**
SiBr4	36.3	-10580.40182	-27778845.0	-27778808.7			-6.9		
SibBr4_F	45.8	-10680.31006	-28041154.1	-28041108.2	608.0	**344.5**	-44.8	155.4	**189.0**
SiI4	34.9	-1478.86721	-3882765.9	-3882730.9			-10.0		
SiI4_F	44.7	-1578.77850	-4145083.0	-4145038.2	600.3	**352.2**	-44.8	168.5	**183.7**
Si(NH2)4	311.0	-512.79916	-1346354.2	-1346043.3			-6.4		
Si(NH2)4_F	318.8	-612.64079	-1608488.4	-1608169.6	781.3	**171.2**	-40.6	170.9	**0.4**
Si(OH)4	176.5	-592.34102	-1555191.4	-1555014.9			-7.1		
Si(OH)4_F	187.9	-692.20359	-1817380.5	-1817192.6	729.9	**222.6**	-43.8	160.7	**62.0**
Si(SH)4	134.8	-1882.64322	-4942879.8	-4942745.0			-8.4		
Si(SH)4_F	144.9	-1982.54363	-5205168.3	-5205023.4	629.2	**323.3**	-43.6	166.5	**156.8**
SiMe4	425.0	-448.52203	-1177594.6	-1177169.6			-3.6		
SiMe4_F	429.9	-548.34828	-1439688.4	-1439258.5	818.7	**133.8**	-35.7	179.8	**-46.0**
SiPh4	1043.4	-1214.19662	-3187873.2	-3186829.9			-13.2		
SiPh4_F	1047.0	-1314.02805	-3449980.6	-3448933.6	803.9	**148.6**	-45.4	179.2	**-30.6**
Si(CCH)4	241.1	-595.61967	-1563799.4	-1563558.3			-8.6		
Si(CCH)4_F	246.4	-695.49230	-1826015.0	-1825768.6	697.3	**255.2**	-53.1	127.7	**127.6**
Si(C2F5)4	371.6	-2588.87031	-6797079.0	-6796707.4			-5.3		
Si(C2F5)4_F	378.1	-2688.80996	-7059470.5	-7059092.4	522.6	**429.9**	-38.2	176.2	**253.7**
Si(C6F5)4	655.0	-3197.33139	-8394593.6	-8393938.6			-13.0		
Si(C6F5)4_F	660.4	-3297.22987	-8656877.0	-8656216.6	616.3	**336.2**	-36.5	215.9	**120.4**
Si(OC6F5)4	711.4	-3498.04554	-9184118.6	-9183407.2			-14.8		
Si(OC6F5)4_F	721.1	-3597.98157	-9446500.6	-9445779.6	521.9	**430.6**	-37.3	219.9	**210.7**
Si(OC(CF3))4	764.1458	-4790.33746	-12577031.0	-12576266.8			-9.0		
Si(OC(CF3))4_F	766.612	-4890.24765	-12839345.2	-12838578.6	582.5	**370.0**	-35.3	204.1	**165.8**
Si(OTf)4	415.9	-4131.50139	-10847256.9	-10846841.0			-12.5		
Si(OTf)4_F	425.4	-4231.47606	-11109740.4	-11109315.0	433.6	**518.9**	-43.2	185.6	**333.3**
Si(N(C6F5)2)4	1375.1	-6328.37649	-16615152.5	-16613777.4			-20.9		
Si(N(C6F5)2)4_F	1380.1	-6428.34311	-16877614.8	-16876234.8	570.1	**382.4**	-39.1	237.8	**144.6**
GeH4	92.6	-2078.05125	-5455923.6	-5455830.9			0.5		
GeH4_F	95.8	-2177.86867	-5717994.2	-5717898.4	840.1	**112.4**	-51.3	97.4	**15.0**
GeF4	44.0	-2474.92812	-6497923.8	-6497879.8			-2.8		
GeF4_F	52.1	-2574.83976	-6760241.8	-6760189.7	597.7	**354.8**	-50.5	114.2	**240.6**
GeCl4	36.6	-3914.87863	-10278513.8	-10278477.3			-5.6		
GeCl4_F	45.4	-4014.77838	-10540800.6	-10540755.3	629.6	**322.9**	-47.0	140.8	**182.1**
GeBr4	34.4	-12366.97787	-32469500.4	-32469466.0			-7.5		
GebBr4_F	43.6	-12466.87710	-32731785.8	-32731742.3	631.4	**321.1**	-47.0	148.5	**172.6**
GeI4	33.5	-3275.59453	-8600073.4	-8600039.9			-10.6		
GeI4_F	42.8	-3375.48527	-8862336.6	-8862293.7	653.8	**298.7**	-47.1	161.4	**137.3**
Ge(NH2)4	306.0	-2299.33181	-6036895.7	-6036589.7			-7.0		
Ge(NH2)4_F	314.3	-2399.17916	-6299044.9	-6298730.6	766.6	**185.9**	-53.9	117.9	**68.0**
Ge(OH)4	171.8	-2378.84465	-6245656.6	-6245484.9			-7.5		
Ge(OH)4_F	182.9	-2478.72049	-6507880.6	-6507697.7	694.8	**257.7**	-55.2	114.4	**143.3**
Ge(SH)4	132.3	-3669.22209	-9633542.6	-9633410.3			-8.6		
Ge(SH)4_F	141.5	-3769.10901	-9895795.7	-9895654.2	663.7	**288.8**	-48.3	148.1	**140.7**
GeMe4	423.5	-2235.10639	-5868271.8	-5867848.3			-3.5		
GeMe4_F	427.0	-2334.91965	-6130331.5	-6129904.5	851.4	**101.1**	-47.8	128.8	**-27.7**
GePh4	1040.5	-3000.77967	-7878547.0	-7877506.5			-13.3		
GePh4_F	1044.1	-3100.58729	-8140591.9	-8139547.8	866.3	**86.2**	-49.3	163.6	**-77.4**
Ge(CCH)4	238.4	-2382.19677	-6254457.6	-6254219.2			-8.6		
Ge(CCH)4_F	243.1	-2482.05025	-6516622.9	-6516379.9	746.9	**205.6**	-55.1	119.4	**86.1**
Ge(C2F5)4	369.3	-4375.47394	-11487806.8	-11487437.6			-5.3		

Ge(C2F5)4_F	374.8	-4475.38062	-11750111.8	-11749737.0	608.2	**344.3**	-38.9	173.6	**170.8**
Ge(C6F5)4	652.2	-4983.91213	-13085261.3	-13084609.1			-13.2		
Ge(C6F5)4_F	657.2	-5083.78830	-13347486.2	-13346829.0	674.3	**278.2**	-39.4	204.5	**73.7**
Ge(OC6F5)4	706.2	-5284.54429	-13874571.0	-13873864.8			-14.7		
Ge(OC6F5)4_F	716.2	-5384.48250	-14136958.8	-14136242.5	516.6	**435.9**	-40.1	207.7	**228.3**
Ge(OC(CF3))4	759.1298	-6576.82968	-17267466.3	-17266707.2			-9.1		
Ge(OC(CF3))4_F	764.6056	-6676.75367	-17529816.8	-17529052.2	549.3	**403.2**	-35.7	203.1	**200.1**
Ge(OTf)4	411.4	-5918.01921	-15537759.4	-15537348.0			-12.4		
Ge(OTf)4_F	423.2	-6017.99655	-15800249.9	-15799826.7	428.9	**523.6**	-43.0	185.8	**337.8**
Ge(N(C6F5)2)4	1371.6	-8115.54324	-21307358.8	-21305987.2			-21.1		
Ge(N(C6F5)2)4_F	1376.7	-8215.50453	-21569807.1	-21568430.5	584.2	**368.3**	-40.1	234.5	**133.8**
SnH4	81.3	-216.31040	-567923.0	-567841.7			0.2		
SnH4_F	86.2	-316.14345	-830034.6	-829948.4	800.8	**151.7**	-28.5	194.1	**-42.4**
SnF4	41.2	-613.16530	-1609865.5	-1609824.3			-5.0		
SnF4_F	49.2	-713.09860	-1872240.4	-1872191.1	540.8	**411.7**	-47.9	134.7	**277.1**
SnCl4	35.5	-2053.21105	-5390705.6	-5390670.1			-6.8		
SnCl4_F	44.2	-2153.12492	-5653029.5	-5652985.3	592.4	**360.1**	-44.8	154.9	**205.2**
SnBr4	33.8	-10542.16472	-27678453.5	-27678419.7			-8.4		
SnBr4_F	42.7	-10642.08005	-27940781.2	-27940738.5	588.8	**363.7**	-44.6	162.8	**200.9**
SnI4	33.1	-1403.76075	-3685573.8	-3685540.7			-11.4		
SnI4_F	42.1	-1503.66364	-3947868.9	-3947826.7	621.6	**330.9**	-44.7	174.8	**156.1**
Sn(NH2)4	302.0	-437.57056	-1148841.5	-1148539.5			-7.8		
Sn(NH2)4_F	309.4	-537.43826	-1411044.1	-1410734.8	712.3	**240.2**	-42.3	169.7	**70.5**
Sn(OH)4	167.6	-517.07732	-1357586.5	-1357418.9			-8.7		
Sn(OH)4_F	177.8	-616.97460	-1619866.8	-1619689.0	637.5	**315.0**	-49.0	145.5	**169.5**
Sn(SH)4	130.4	-1807.54434	-4745707.7	-4745577.3			-9.4		
Sn(SH)4_F	139.2	-1907.44209	-5007989.2	-5007850.0	634.9	**317.6**	-45.0	165.2	**152.3**
SnMe4	420.2	-373.36021	-980257.2	-979837.0			-4.0		
SnMe4_F	422.9	-473.19295	-1242368.1	-1241945.2	799.4	**153.1**	-34.0	188.4	**-35.4**
SnPh4	1037.3	-1139.04899	-2990573.1	-2989535.8			-14.1		
SnPh4_F	1042.1	-1238.89428	-3252716.9	-3251674.8	768.6	**183.9**	-45.3	183.1	**0.8**
Sn(CCH)4	235.9	-520.45543	-1366455.7	-1366219.8			-9.4		
Sn(CCH)4_F	240.6	-620.32441	-1628661.7	-1628421.1	706.3	**246.2**	-51.9	136.3	**109.9**
Sn(C2F5)4	366.9	-2513.73926	-6599822.4	-6599455.5			-5.7		
Sn(C2F5)4_F	372.6	-2613.65352	-6862147.3	-6861774.8	588.4	**364.1**	-37.4	181.3	**182.9**
Sn(C6F5)4	649.7	-3124.16919	-8202506.2	-8201856.5			-13.6		
Sn(C6F5)4_F	654.9	-3224.11688	-8464918.9	-8464264.0	620.0	**332.5**	-36.4	218.7	**113.8**
Sn(OC6F5)4	702.7	-3424.96983	-8992258.3	-8991555.6			-15.2		
Sn(OC6F5)4_F	710.6	-3524.97914	-9254832.7	-9254122.2	460.9	**491.6**	-39.3	213.0	**278.6**
Sn(OC(CF3))4	753.6122	-4717.78679	-12386549.2	-12385795.6			-9.5		
Sn(OC(CF3))4_F	760.0494	-4817.78515	-12649094.9	-12648334.9	488.2	**464.3**	-35.4	205.6	**258.7**
Sn(OTf)4	411.1	-4056.34295	-10649928.4	-10649517.3			-13.2		
Sn(OTf)4_F	420.9	-4156.31779	-10912412.4	-10911991.4	433.5	**519.0**	-42.3	192.2	**326.9**
PH5	124.9	-343.81959	-902698.3	-902573.5			-1.4		
PH5_F	136.9	-443.66862	-1164852.0	-1164715.0	766.1	**186.4**	-52.5	100.4	**86.1**
PF5	61.8	-839.96198	-2205320.2	-2205258.4			-1.8		
PF5_F	69.9	-939.88476	-2467667.4	-2467597.5	568.5	**384.0**	-51.0	108.3	**275.7**
PCl5	46.9	-2639.78434	-6930753.8	-6930706.9			-6.2		
PCl5_F	56.6	-2739.71242	-7193115.0	-7193058.3	556.2	**396.3**	-46.6	145.0	**251.3**
PBr5	43.3	-13204.89922	-34669462.9	-34669419.6			-8.7		
PBr5_F	53.5	-13304.83116	-34931834.2	-34931780.7	546.5	**406.0**	-46.7	155.1	**250.9**
PI5	41.8	-1827.99907	-4799411.6	-4799369.7			-12.7		
PI5_F	52.1	-1927.92528	-5061767.8	-5061715.7	561.7	**390.8**	-47.2	169.8	**221.1**
P(NH2)5	396.2	-620.46017	-1629018.2	-1628621.9			-7.7		
P(NH2)5_F	403.8	-720.27647	-1891085.9	-1890682.0	847.5	**105.0**	-52.6	126.2	**-21.2**
P(OH)5	228.9	-719.86945	-1890017.2	-1889788.4			-7.0		
P(OH)5_F	234.4	-819.71279	-2152155.9	-2151921.5	774.5	**178.0**	-56.9	105.3	**72.7**
P(SH)5	170.1	-2332.76043	-6124662.5	-6124492.4			-9.0		
P(SH)5_F	177.9	-2432.63865	-6386892.8	-6386714.9	685.1	**267.4**	-47.0	155.0	**112.4**
PMe5	537.1	-540.13369	-1418121.0	-1417583.9			-4.1		
PMe5_F	542.5	-639.96449	-1680226.8	-1679684.2	807.2	**145.3**	-45.3	141.8	**3.5**
PPh5	1303.4	-1498.43855	-3934150.4	-3932847.0			-15.6		
PPh5_F	1310.1	-1598.34619	-4196457.9	-4195147.9	726.6	**225.9**	-47.2	181.6	**44.4**
P(CCH)5	300.4	-723.96977	-1900782.6	-1900482.2			-10.3		
P(CCH)5_F	307.6	-823.86065	-2163046.1	-2162738.6	651.3	**301.2**	-55.9	123.6	**177.6**
P(C2F5)5	463.4	-3215.52283	-8442355.2	-8441891.8			-6.4		
P(C2F5)5_F	471.1	-3315.49132	-8704822.4	-8704351.4	448.1	**504.4**	-37.3	184.5	**320.0**
P(C6F5)5	816.6	-3976.09874	-10439247.2	-10438430.6			-14.8		
P(C6F5)5_F	823.2	-4075.99701	-10701530.2	-10700707.0	617.9	**334.6**	-37.7	218.3	**116.3**
P(OC6F5)5	889.9	-4351.97554	-11426111.8	-11425221.9			-16.3		
P(OC6F5)5_F	899.0	-4451.91985	-11688515.6	-11687616.6	499.6	**452.9**	-39.5	216.7	**236.2**
P(OC(CF3))5	946.2684	-5967.16185	-15666783.4	-15665837.2			-10.1		
P(OC(CF3))5_F	953.04	-6067.11680	-15929215.2	-15928262.1	469.3	**483.2**	-34.8	210.8	**272.4**
P(OTf)5	520.9534	-5143.77129	-13504971.5	-13504450.6			-13.5		
P(OTf)5_F	531.8214	-5243.76082	-13767494.0	-13766962.2	396.0	**556.5**	-42.7	191.7	**364.9**
AsH5	115.0	-2237.40186	-5874298.6	-5874183.6			-1.2		
AsH5_F	124.9	-2337.24233	-6136429.7	-6136304.9	786.4	**166.1**	-50.9	105.9	**60.2**
AsF5	55.5	-2733.48473	-7176764.2	-7176708.6			-2.4		
AsF5_F	63.8	-2833.42848	-7439166.5	-7439102.7	513.6	**438.9**	-49.8	115.6	**323.4**
AsCl5	44.6	-4533.39021	-11902416.0	-11902371.4			-6.4		
AsCl5_F	54.0	-4633.31975	-12164781.0	-12164727.1	551.9	**400.6**	-46.5	146.2	**254.3**
AsBr5	41.8	-15098.51787	-39641158.7	-39641116.8			-8.8		
AsBr5_F	51.5	-15198.44448	-39903516.0	-39903464.5	559.9	**392.6**	-46.9	154.5	**238.1**
AsI5	40.8	-3728.91371	-9790262.9	-9790222.1			-12.6		
AsI5_F	50.5	-3828.82824	-10052588.5	-10052538.1	591.6	**360.9**	-47.3	169.1	**191.8**
As(NH2)5	388.2	-2514.01493	-6600546.2	-6600158.0			-8.1		
As(NH2)5_F	395.3	-2613.85495	-6862676.2	-6862280.9	784.8	**167.7**	-52.4	128.9	**38.8**
As(OH)5	220.2	-2613.40632	-6861498.3	-6861278.1			-7.4		
As(OH)5_F	226.4	-2713.27429	-7123701.6	-7123475.2	710.5	**242.0**	-56.3	109.4	**132.6**
As(SH)5	166.5	-4226.36451	-11096320.0	-11096153.5			-9.3		

As(SH)5_F	174.2	-4326.24610	-11358559.1	-11358384.9	676.2	**276.3**	-47.1	156.2	**120.0**
AsMe5	530.2	-2433.71772	-6389725.9	-6389195.7			-4.3		
AsMe5_F	534.5	-2533.55195	-6651840.7	-6651306.1	797.1	**155.4**	-45.6	141.4	**13.9**
AsPh5	1299.1	-3392.64604	-8907392.2	-8906093.1			-15.8		
AsPh5_F	1305.2	-3492.55377	-9169699.9	-9168394.7	725.9	**226.6**	-48.3	178.0	**48.7**
As(CCH)5	296.5	-2617.55107	-6872380.3	-6872083.8			-11.5		
As(CCH)5_F	303.2	-2717.43590	-7134628.0	-7134324.7	666.7	**285.8**	-56.2	127.0	**158.8**
As(C2F5)5	460.1	-5109.16369	-13414109.3	-13413649.1			-6.6		
As(C2F5)5_F	466.7	-5209.09145	-13676469.6	-13676002.9	553.9	**398.6**	-37.6	184.4	**214.2**
As(C6F5)5	813.2	-5869.69662	-15410888.5	-15410075.3			-15.1		
As(C6F5)5_F	819.5	-5969.59133	-15673162.0	-15672342.5	627.0	**325.5**	-38.2	217.5	**108.0**
As(OC6F5)5	885.3	-6245.50947	-16397585.1	-16396699.8			-16.6		
As(OC6F5)5_F	893.2	-6345.47468	-16660043.8	-16659150.6	443.5	**509.0**	-39.9	216.3	**292.7**
As(OC(CF3))5	942.2138	-7860.74065	-20638374.6	-20637432.4			-10.3		
As(OC(CF3))5_F	949.1944	-7960.70352	-20900827.1	-20899877.9	448.8	**503.7**	-35.0	210.6	**293.1**
As(OTf)5	515.3	-7037.32335	-18476492.5	-18475977.2			-13.8		
As(OtTf)5_F	525.9	-7137.32848	-18739055.9	-18738530.0	354.8	**597.7**	-42.5	193.7	**404.0**
SbH5	102.9	-242.77354	-637401.9	-637299.0			-1.5		
SbH5_F	111.0	-342.63349	-899584.2	-899473.2	733.4	**219.1**	-47.8	120.1	**99.0**
SbF5	52.0828	-738.87245	-1939909.6	-1939857.5			-4.6		
SbF5_F	60.3174	-838.83775	-2202368.5	-2202308.2	457.0	**495.5**	-47.7	133.7	**361.9**
SbCl5	43.8	-2538.87720	-6665822.1	-6665778.3			-7.2		
SbCl5_F	52.9	-2638.82109	-6928224.8	-6928171.8	514.1	**438.4**	-45.6	153.3	**285.1**
SbBr5	41.4	-13150.07107	-34525511.6	-34525470.2			-9.4		
SbBr5_F	50.7	-13250.00537	-34787889.1	-34787838.4	539.4	**413.1**	-46.4	159.2	**254.0**
SbI5	40.6	-1727.06082	-4534398.2	-4534357.6			-13.1		
SbI5_F	49.9	-1826.99013	-4796762.6	-4796712.7	552.5	**400.0**	-47.4	170.3	**229.7**
Sb(NH2)5	379.8	-519.38483	-1363644.9	-1363265.1			-9.3		
Sb(NH2)5_F	388.4	-619.25568	-1625855.8	-1625467.4	705.3	**247.2**	-51.9	135.8	**111.4**
Sb(OH)5	213.7	-618.77939	-1624605.3	-1624391.6			-9.0		
Sb(OH)5_F	221.2	-718.67792	-1886888.9	-1886667.7	631.5	**321.0**	-55.0	121.6	**199.4**
Sb(SH)5	164.1	-2231.82141	-5859647.1	-5859483.0			-10.0		
Sb(SH)5_F	172.1	-2331.72413	-6121941.7	-6121769.6	621.0	**331.5**	-46.7	160.1	**171.4**
SbMe5	524.4	-439.09807	-1152852.0	-1152327.6			-4.8		
SbMe5_F	525.7	-538.95268	-1415020.3	-1414494.6	740.6	**211.9**	-45.3	144.3	**67.6**
SbPh5	1296.2	-1397.28998	-3668584.8	-3667288.7			-16.0		
SbPh5_F	1301.4	-1497.21927	-3930949.2	-3929647.7	668.4	**284.1**	-49.2	174.9	**109.2**
Sb(CCH)5	293.7	-622.94023	-1635529.6	-1635235.9			-11.5		
Sb(CCH)5_F	299.8	-722.84141	-1897820.1	-1897520.3	623.2	**329.3**	-56.3	126.5	**202.8**
Sb(C2F5)5	457.7	-3114.55892	-8177274.5	-8176816.8			-6.7		
Sb(C2F5)5_F	464.3	-3214.50124	-8439673.0	-8439208.7	515.7	**436.8**	-37.7	184.3	**252.5**
Sb(C6F5)5	811.1	-3877.55579	-10180522.7	-10179711.6			-15.8		
Sb(C6F5)_F	817.4	-3977.52348	-10442987.9	-10442170.5	568.6	**383.9**	-38.9	217.0	**166.9**
Sb(OC6F5)5	879.1	-4253.59350	-11167809.7	-11166930.6			-16.8		
Sb(OC6F5)5_F	890.1	-4353.62353	-11430438.6	-11429548.5	409.6	**542.9**	-40.2	216.3	**326.6**
Sb(OC(CF3))5	941.545	-5869.52591	-15410440.3	-15409498.7			-9.7		
Sb(OC(CF3))5_F	946.6028	-5969.55980	-15673079.3	-15672132.7	393.5	**559.0**	-37.8	196.4	**362.6**
Sb(OTf)5	512.9	-5042.74439	-13239725.4	-13239212.5			-14.8		
Sb(OtTf)5_F	523.5	-5142.74813	-13502285.2	-13501761.7	358.4	**594.1**	-42.7	197.0	**397.2**
Ox3									
PH3	75.6998	-342.70935	-899783.4	-899707.7			-1.1		
PH3_F	82.1788	-442.50927	-1161808.1	-1161725.9	889.4	**63.1**	-54.8	89.3	**-26.2**
PF3	36.1152	-640.35404	-1681249.5	-1681213.4			-1.7		
PF3_F	43.5138	-740.20631	-1943411.7	-1943368.2	752.9	**199.6**	-52.6	100.9	**98.7**
PCl3	29.0928	-1720.27177	-4516573.5	-4516544.4			-4.3		
PCl3_F	37.7454	-1820.14924	-4778801.8	-4778764.1	688.0	**264.5**	-49.9	123.2	**141.4**
PBr3	27.2954	-8059.34160	-21159801.4	-21159774.1			-5.8		
PBr3_F	36.5332	-8159.22741	-21422051.6	-21422015.0	666.6	**285.9**	-50.1	128.4	**157.5**
P(C2F5)3	280.7706	-2065.75687	-5423644.7	-5423363.9			-4.5		
P(C2F5)3_F	286.0792	-2165.63670	-5685879.2	-5685593.1	678.4	**274.1**	-40.5	163.2	**110.9**
P(OC(CF3))3	571.4478	-3716.76527	-9758367.2	-9757795.8			-7.6		
P(OC(CF3))3_F	576.5056	-3816.65698	-10020632.9	-10020056.4	633.7	**318.8**	-36.7	192.4	**126.5**
AsH3	70.6002	-2236.31330	-5871440.6	-5871370.0			-10.3		
AsH3_F	76.4104	-2336.11720	-6133475.7	-6133399.3	878.3	**74.2**	-52.7	136.4	**-62.2**
AsF3	32.8966	-2533.93586	-6652848.6	-6652815.7			-3.2		
AsF3_F	40.4206	-2633.80498	-6915055.0	-6915014.6	708.7	**243.8**	-52.2	109.1	**134.7**
AsCl3	27.5462	-3613.89140	-9488271.9	-9488244.3			-5.0		
AsCl3_F	35.948	-3713.77325	-9750511.7	-9750475.7	676.2	**276.3**	-49.0	130.0	**146.3**
AsBr3	26.2086	-9952.96780	-26131517.0	-26131490.8			-6.2		
AsBr3_F	34.9866	-10052.85334	-26393766.4	-26393731.5	666.9	**285.6**	-48.8	136.1	**149.5**
As(C2F5)3	277.761	-3959.37077	-10395328.0	-10395050.2			-4.6		
As(C2F5)3_F	283.2786	-4059.24812	-10657555.9	-10657272.7	685.2	**267.3**	-39.5	167.8	**99.5**
As(OC(CF3))3	568.3546	-5610.35740	-14729993.4	-14729425.0			-10.3		
As(OC(CF3))3_F	573.3288	-5710.25743	-14992280.9	-14991707.6	611.7	**340.8**	-37.0	202.4	**138.4**
SbH3	62.5746	-241.68416	-634541.8	-634479.2			0.5		
SbH3_F	68.4266	-341.50891	-896631.7	-896563.2	823.6	**128.9**	-49.1	106.5	**22.4**
SbF3	31.2246	-539.34091	-1416039.6	-1416008.3			-6.0		
SbF3_F	38.9576	-639.22758	-1678292.0	-1678253.1	662.9	**289.6**	-51.6	123.1	**166.5**
SbCl3	27.0446	-1619.31369	-4251508.1	-4251481.0			-6.8		
SbCl3_F	35.3628	-1719.20793	-4513780.4	-4513745.1	643.6	**308.9**	-48.3	140.3	**168.6**
SbBr3	25.8324	-7958.39073	-20894754.9	-20894729.0			-7.6		
SbBr3_F	34.4432	-8058.28723	-21157033.1	-21156998.7	638.0	**314.5**	-47.9	145.3	**169.2**
Sb(C2F5)3	275.9218	-1964.75386	-5158461.3	-5158185.3			-5.4		
Sb(C2F5)3_F	281.3976	-2064.64934	-5420736.8	-5420455.4	637.5	**315.0**	-39.2	172.8	**142.1**
Sb(OC(CF3))3	566.599	-3617.82782	-9498606.9	-9498040.3			-10.7		
Sb(OC(CF3))3_F	572.2002	-3717.80201	-9761089.2	-9760517.0	550.8	**401.7**	-35.2	211.2	**190.4**
Ox4									
SH4	103.5804	-400.01719	-1050245.1	-1050141.5			-1.2		
SH4_F	109.6832	-499.80327	-1312233.5	-1312123.8	925.4	**27.1**	-61.1	63.3	**-36.2**
SF4	47.1086	-796.78978	-2091971.6	-2091924.5			-2.1		

SF4_F	53.6294	-896.65070	-2354156.4	-2354102.8	729.3	**223.2**	-51.9	105.7	**117.5**
S(C2F5)4	371.5602	-2697.40929	-7082048.1	-7081676.5			-5.7		
S(C2F5)4_F	375.6984	-2797.24740	-7344173.1	-7343797.4	786.8	**165.7**	-38.1	178.6	**-12.9**
S(OC(CF3))4	757.3742	-4898.67427	-12861469.3	-12860711.9			-9.0		
S(OC(CF3))4_F	762.5992	-4998.56592	-13123734.8	-13122972.2	634.0	**318.5**	-35.8	201.8	**116.7**
SeH4	93.841	-2402.44995	-6307632.4	-6307538.5			0.1		
SeH4_F	98.1046	-2502.23793	-6569625.7	-6569527.6	918.5	**34.0**	-52.8	92.4	**-58.5**
SeF4	43.1376	-2799.25620	-7349447.1	-7349404.0			-3.7		
SeF4_F	50.369	-2899.14353	-7611701.3	-7611651.0	660.6	**291.9**	-51.3	115.0	**176.9**
Se(C2F5)4	368.7178	-4699.86733	-12339501.7	-12339133.0			-5.9		
Se(C2F5)4_F	373.5666	-4799.73047	-12601692.3	-12601318.8	721.8	**230.7**	-38.0	179.9	**50.8**
Se(OC(CF3))4	754.5736	-6901.14652	-18118960.2	-18118205.6			-9.2		
Se(OC(CF3))4_F	760.342	-7001.05655	-18381274.0	-18380513.6	586.3	**366.2**	-35.5	204.1	**162.1**
TeH4	83.7672	-269.97162	-708810.5	-708726.7			-0.4		
TeH4_F	87.2784	-369.78873	-970880.3	-970793.0	841.3	**111.2**	-49.0	110.5	**0.7**
TeF4	41.4238	-666.84304	-1750796.4	-1750755.0			-6.3		
TeF4_F	48.906	-766.75184	-2013106.9	-2013058.0	604.5	**348.0**	-50.6	128.8	**219.1**
Te(C2F5)4	366.7532	-2567.39137	-6740686.0	-6740319.3			-6.6		
Te(C2F5)4_F	372.3544	-2667.30391	-7003006.4	-7002634.1	592.8	**359.7**	-38.1	181.9	**177.8**
Te(OC(CF3))4	753.6958	-4771.42671	-12527380.8	-12526627.1			-9.7		
Te(OC(CF3))4_F	759.8404	-4871.41173	-12789891.5	-12789131.7	522.9	**429.6**	-35.8	204.7	**224.9**
Special Acids									
B(C6F4-pCF3)3	624.1158	-2920.61801	-7668082.6	-7667458.5			-12.7		
B(C6F4-pCF3)3_F	631.4726	-3020.62520	-7930651.5	-7930020.0	465.9	**486.6**	-37.7	209.5	**277.0**
B(OTeF5)3	224.1734	-2551.45028	-6698832.7	-6698608.5			-8.1		
B(OTeF5)3_F	228.1444	-2651.46356	-6961417.6	-6961189.4	446.6	**505.9**	-37.2	192.2	**313.7**
B(OC5F4N)3	509.8346	-2183.62820	-5733115.8	-5732606.0			-13.3		
B(OC5F4N)3_F	515.394	-2283.62542	-5995658.5	-5995143.2	490.3	**462.2**	-39.2	205.9	**256.3**
F4C6(1,2-B(C6F5)2)2	828.0998	-3587.48528	-9418942.6	-9418114.5			-14.8		
F4C6(1,2-B(C6F5)2)2_F	835.5402	-3687.49803	-9681526.1	-9680690.5	451.4	**501.1**	-37.7	218.4	**282.7**
perfluoroborole	898.4492	-3814.71597	-10015536.8	-10014638.3			-16.6		
perfluoroborole_F	905.4716	-3914.72109	-10278100.2	-10277194.8	471.0	**481.5**	-41.0	211.9	**269.6**
F4C6(1,2-(B(C12F8))2	787.8464	-3188.26217	-8370782.3	-8369994.5			-13.3		
F4C6(1,2-(B(C12F8))2_F	790.3126	-3288.26779	-8633347.1	-8632556.8	465.2	**487.3**	-37.9	211.2	**276.1**
Al(OTeF5)3	213.5562	-2768.94934	-7269876.5	-7269662.9			-8.3		
Al(OTeF5)3_F	220.495	-2868.99890	-7532556.6	-7532336.1	354.3	**598.2**	-37.3	192.6	**405.6**
Al(OC5F4N)3	497.838	-2401.10878	-6304111.1	-6303613.3			-15.6		
Al(OC5F4N)3_F	504.4006	-2501.15678	-6566787.1	-6566282.7	358.0	**594.5**	-38.8	216.8	**377.7**
Al(OC(C6F5)3)3	1564.574	-7129.87190	-18719478.7	-18717914.1			-23.8		
Al(OC(C6F5)3)3_F	1574.4388	-7229.90008	-18982102.7	-18980528.2	413.3	**539.2**	-41.9	238.2	**301.0**
As(OTeF5)5	350.1168	-6446.21142	-16924528.1	-16924178.0			-9.7		
As(OTeF5)5_F	360.943	-6546.25550	-17187193.8	-17186832.9	372.6	**579.9**	-35.5	206.2	**373.7**
Sb(OTeF5)5	350.0332	-4450.88550	-11685799.9	-11685449.8			-10.0		
Sb(OTeF5)5_F	358.6022	-4550.94517	-11948506.5	-11948147.9	329.4	**623.1**	-35.6	206.8	**416.3**

Comparisons

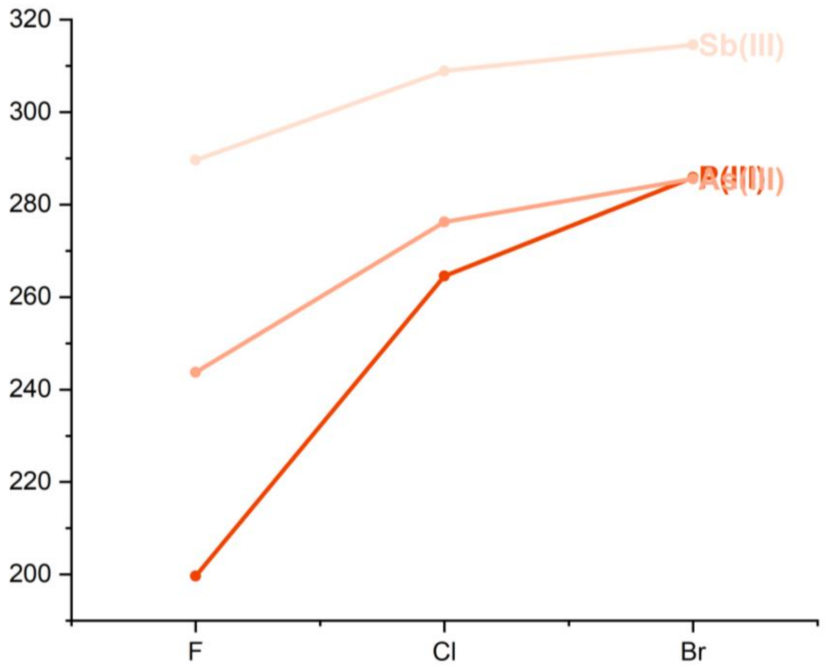

Figure S1: Comparisons of computed FIA for group 15(III) halides.

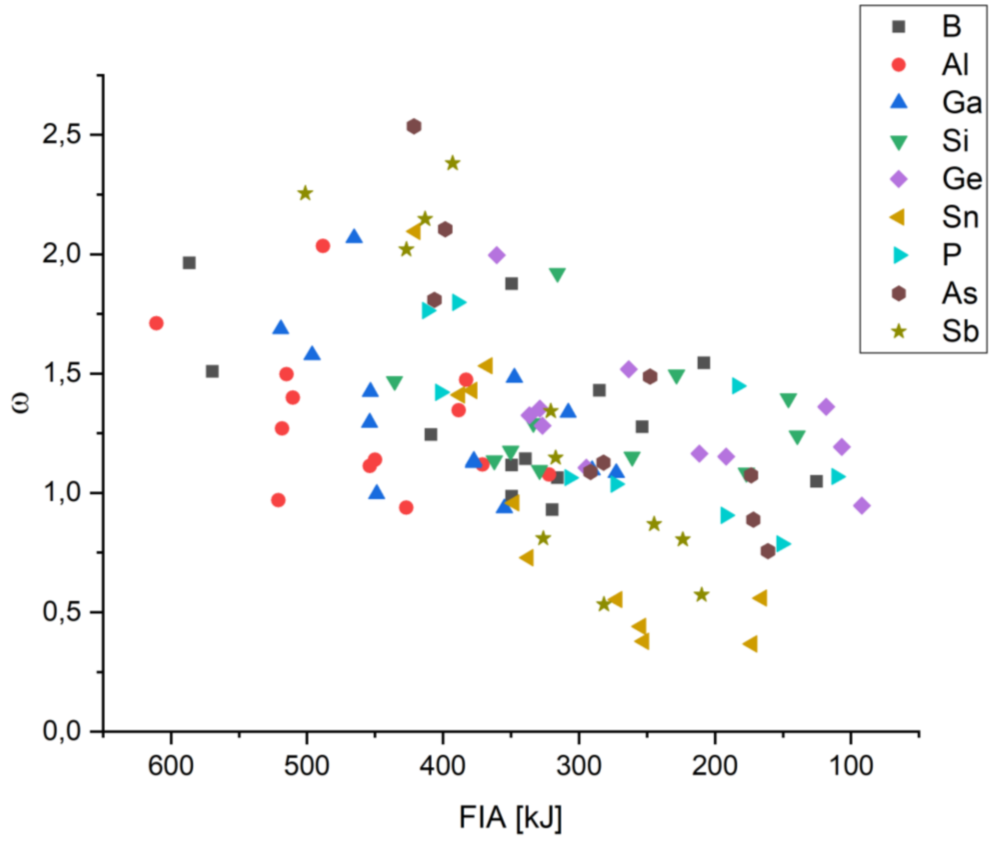

Figure S2: Comparisons of computed FIA with the global electrophilicity index, obtained from the PBEh-3c HOMO/LUMO energies.

Literature

[1] F. Neese, *Wires. Comput. Mol. Sci.* **2012**, *2*, 73-78.
[2] S. Grimme, J. G. Brandenburg, C. Bannwarth, A. Hansen, *J. Chem. Phys.* **2015**, *143*, 054107.
[3] S. Grimme, *Chem-Eur. J.* **2012**, *18*, 9955-9964.
[4] G. D. P. III, R. J. Bartlett, *J. Chem. Phys.* **1982**, *76*, 1910-1918.
[5] T. J. Lee, P. R. Taylor, *Int. J. Quantum Chem* **1989**, *36*, 199-207.
[6] a) R. A. Kendall, T. H. Dunning, R. J. Harrison, *J. Chem. Phys.* **1992**, *96*, 6796-6806; b) D. E. Woon, T. H. Dunning, *J. Chem. Phys.* **1993**, *98*, 1358-1371; c) A. K. Wilson, D. E. Woon, K. A. Peterson, T. H. D. Jr., *J. Chem. Phys.* **1999**, *110*, 7667-7676.
[7] a) B. Metz, H. Stoll, M. Dolg, *J. Chem. Phys.* **2000**, *113*, 2563-2569; b) K. A. Peterson, K. E. Yousaf, *J. Chem. Phys.* **2010**, *133*, 174116.
[8] a) T. Helgaker, W. Klopper, H. Koch, J. Noga, *J. Chem. Phys.* **1997**, *106*, 9639-9646; b) A. Halkier, T. Helgaker, P. Jørgensen, W. Klopper, H. Koch, J. Olsen, A. K. Wilson, *Chem. Phys. Lett.* **1998**, *286*, 243-252; c) F. Neese, E. F. Valeev, *J. Chem. Theo. Comp.* **2011**, *7*, 33-43; d) D. G. Liakos, F. Neese, *J. Phys. Chem. A* **2012**, *116*, 4801-4816.
[9] J. Friedrich, E. Perlt, M. Roatsch, C. Spickermann, B. Kirchner, *J. Chem. Theo. Comp.* **2011**, *7*, 843-851.
[10] M. J. Frisch, G. W. Trucks, H. B. Schlegel, G. E. Scuseria, M. A. Robb, J. R. Cheeseman, G. Scalmani, V. Barone, G. A. Petersson, H. Nakatsuji, X. Li, M. Caricato, A. V. Marenich, J. Bloino, B. G. Janesko, R. Gomperts, B. Mennucci, H. P. Hratchian, J. V. Ortiz, A. F. Izmaylov, J. L. Sonnenberg, Williams, F. Ding, F. Lipparini, F. Egidi, J. Goings, B. Peng, A. Petrone, T. Henderson, D. Ranasinghe, V. G. Zakrzewski, J. Gao, N. Rega, G. Zheng, W. Liang, M. Hada, M. Ehara, K. Toyota, R. Fukuda, J. Hasegawa, M. Ishida, T. Nakajima, Y. Honda, O. Kitao, H. Nakai,

T. Vreven, K. Throssell, J. A. Montgomery Jr., J. E. Peralta, F. Ogliaro, M. J. Bearpark, J. J. Heyd, E. N. Brothers, K. N. Kudin, V. N. Staroverov, T. A. Keith, R. Kobayashi, J. Normand, K. Raghavachari, A. P. Rendell, J. C. Burant, S. S. Iyengar, J. Tomasi, M. Cossi, J. M. Millam, M. Klene, C. Adamo, R. Cammi, J. W. Ochterski, R. L. Martin, K. Morokuma, O. Farkas, J. B. Foresman, D. J. Fox, Wallingford, CT, **2016**.

[11] L. A. Curtiss, P. C. Redfern, K. Raghavachari, *J. Chem. Phys.* **2007**, *126*, 084108.

[12] a) F. Neese, A. Hansen, D. G. Liakos, *J. Chem. Phys.* **2009**, *131*; b) C. Riplinger, F. Neese, *J. Chem. Phys.* **2013**, *138*; c) C. Riplinger, B. Sandhoefer, A. Hansen, F. Neese, *J. Chem. Phys.* **2013**, *139*.

[13] F. Neese, F. Wennmohs, A. Hansen, U. Becker, *Chem. Phys.* **2009**, *356*, 98-109.

[14] a) J. P. Perdew, *Phys. Rev. B* **1986**, *33*, 8822-8824; b) A. D. Becke, *Phys. Rev. A* **1988**, *38*, 3098-3100.

[15] a) S. Grimme, J. Antony, S. Ehrlich, H. Krieg, *J. Chem. Phys.* **2010**, *132*, 154104; b) S. Grimme, J. Antony, S. Ehrlich, H. Krieg, *J. Chem. Phys.* **2010**, *132*; c) S. Grimme, S. Ehrlich, L. Goerigk, *J. Comput. Chem.* **2011**, *32*, 1456-1465; d) E. R. Johnson, A. D. Becke, *J. Chem. Phys.* **2005**, *123*; e) A. D. Becke, E. R. Johnson, *J. Chem. Phys.* **2005**, *122*.

[16] A. Schafer, C. Huber, R. Ahlrichs, *J. Chem. Phys.* **1994**, *100*, 5829-5835.

[17] A. D. Becke, *J. Chem. Phys.* **1993**, *98*, 5648-5652.

[18] Y. Zhao, D. G. Truhlar, *J. Phys. Chem. A* **2005**, *109*, 5656-5667.

[19] a) F. Weigend, F. Furche, R. Ahlrichs, *J. Chem. Phys.* **2003**, *119*, 12753-12762; b) F. Weigend, R. Ahlrichs, *Phys. Chem. Chem. Phys.* **2005**, *7*, 3297-3305.

[20] Y. Zhao, D. G. Truhlar, *Theor. Chem. Acc.* **2008**, *120*, 215-241.

[21] S. Grimme, *J. Chem. Phys.* **2006**, *124*, 034108.

[22] S. Kozuch, D. Gruzman, J. M. L. Martin, *J. Phys. Chem. C.* **2010**, *114*, 20801-20808.

[23] S. Kozuch, J. M. L. Martin, *J. Comput. Chem.* **2013**, *34*, 2327-2344.

[24] S. Kozuch, J. M. L. Martin, *Phys. Chem. Chem. Phys.* **2011**, *13*, 20104-20107.

[25] K. Eichkorn, O. Treutler, H. Ohm, M. Haser, R. Ahlrichs, *Chem. Phys. Lett.* **1995**, *242*, 652-660.

[26] K. Eichkorn, F. Weigend, O. Treutler, R. Ahlrichs, *Theor. Chem. Acc.* **1997**, *97*, 119-124.

[27] a) J. Tomasi, B. Mennucci, R. Cammi, *Chem. Rev.* **2005**, *105*, 2999-3094; b) A. Klamt, B. Mennucci, J. Tomasi, V. Barone, C. Curutchet, M. Orozco, F. J. Luque, *Acc. Chem. Res.* **2009**, *42*, 489-492.

[28] a) E. J. Baerends, T. Ziegler, A. J. Atkins, J. Autschbach, D. Bashford, O. Baseggio, A. Brces, F. M. Bickelhaupt, C. Bo, P. M. Boerritger, L. Cavallo, C. Daul, D. P. Chong, D. V. Chulhai, L. Deng, R. M. Dickson, J. M. Dieterich, D. E. Ellis, M. van Faassen, A. Ghysels, A. Giammona, S. J. A. van Gisbergen, A. Goez, A. W. Gtz, S. Gusarov, F. E. Harris, P. van den Hoek, Z. Hu, C. R. Jacob, H. Jacobsen, L. Jensen, L. Joubert, J. W. Kaminski, G. van Kessel, C. Knig, F. Kootstra, A. Kovalenko, M. Krykunov, E. van Lenthe, D. A. McCormack, A. Michalak, M. Mitoraj, S. M. Morton, J. Neugebauer, V. P. Nicu, L. Noodleman, V. P. Osinga, S. Patchkovskii, M. Pavanello, C. A. Peeples, P. H. T. Philipsen, D. Post, C. C. Pye, H. Ramanantoanina, P. Ramos, W. Ravenek, J. I. Rodrguez, P. Ros, R. Rger, P. R. T. Schipper, D. Schlns, H. van Schoot, G. Schreckenbach, J. S. Seldenthuis, M. Seth, J. G. Snijders, Sol; b) C. C. Pye, T. Ziegler, E. van Lenthe, J. N. Louwen, *Can. J. Chem.* **2009**, *87*, 790-797.

[29] E. Van Lenthe, E. J. Baerends, *J. Comput. Chem.* **2003**, *24*, 1142-1156.

[30] a) F. Eckert, A. Klamt, *AlChE J.* **2002**, *48*, 369-385; b) A. Klamt, M. Diedenhofen, *J. Comput. Aided Mol. Des.* **2010**, *24*, 357-360; c) A. Klamt, *J. Phys. Chem.* **1995**, *99*, 2224-2235; d) A. Klamt, B. Mennucci, J. Tomasi, V. Barone, C. Curutchet, M. Orozco, F. J. Luque, *Acc. Chem. Res.* **2009**, *42*, 489-492.

[31] R. Franke, B. Hannebauer, *Phys. Chem. Chem. Phys.* **2011**, *13*, 21344-21350.

[32] F. Eckert, I. Leito, I. Kaljurand, A. Kütt, A. Klamt, M. Diedenhofen, *J. Comput. Chem.* **2009**, *30*, 799-810.

[33] a) A. Ben-Naim, Y. Marcus, *J. Chem. Phys.* **1984**, *81*, 2016-2027; b) D. M. Camaioni, C. A. Schwerdtfeger, *J. Phys. Chem. A* **2005**, *109*, 10795-10797.
